# Genetic Map Construction and Fiber Quality QTL Mapping Using the CottonSNP80K Array in Upland Cotton

**DOI:** 10.3389/fpls.2018.00225

**Published:** 2018-02-27

**Authors:** Zhaoyun Tan, Zhiqin Zhang, Xujing Sun, Qianqian Li, Ying Sun, Peng Yang, Wenwen Wang, Xueying Liu, Chunling Chen, Dexing Liu, Zhonghua Teng, Kai Guo, Jian Zhang, Dajun Liu, Zhengsheng Zhang

**Affiliations:** College of Agronomy and Biotechnology, Southwest University, Chongqing, China

**Keywords:** upland cotton, CottonSNP80K assay, genetic map, fiber quality traits, QTL

## Abstract

Cotton fiber quality traits are controlled by multiple quantitative trait loci (QTL), and the improvement of these traits requires extensive germplasm. Herein, an Upland cotton cultivar from America, Acala Maxxa, was crossed with a local high fiber quality cultivar, Yumian 1, and 180 recombinant inbred lines (RILs) were obtained. In order to dissect the genetic basis of fiber quality differences between these parents, a genetic map containing 12116 SNP markers was constructed using the CottonSNP80K assay, which covered 3741.81 cM with an average distance of 0.31 cM between markers. Based on the genetic map and growouts in three environments, we detected a total of 104 QTL controlling fiber quality traits. Among these QTL, 25 were detected in all three environments and 35 in two environments. Meanwhile, 19 QTL clusters were also identified, and nine contained at least one stable QTL (detected in three environments for a given trait). These stable QTL or QTL clusters are priorities for fine mapping, identifying candidate genes, elaborating molecular mechanisms of fiber development, and application in cotton breeding programs by marker-assisted selection (MAS).

## Introduction

*Gossypium hirsutum* L. (2*n* = 4*x* = 52), one of the 52 *Gossypium* species, is the most important natural fiber crop and yields ~95% of all cotton fiber due to its high productivity and wide adaptability (Chen et al., 2007). However, its moderate fiber qualities for spinning, especially length, strength, and fineness (“micronaire”), are in urgent need of improvement. And the most effective methods may be integrate traditional breeding and the high-efficiency method known as marker-assisted selection (MAS).

Cotton fiber quality traits are controlled by multiple quantitative trait loci (QTL) and affected greatly by environment, which leads to inefficient selection of traditional breeding approaches. However, MAS can differentiate homozygous individuals from heterozygotes and eliminate environmental effects by selection based on DNA marker genotype, promoting breeding progress and shortening breeding cycles. Unfortunately, large gaps still exist between the expectations and actual applications in practical plant breeding (Xu and Crouch, 2008), because of the limited numbers of established molecular markers linked to traits prioritized by breeders (Yang et al., 2015). In cotton, although approximately 1506 QTL controlling fiber quality traits have been identified according to CottonQTLdb (Said et al., 2013, 2015a,b), the majority explained small effects or were unstable in different environments (Li C. et al., 2016; Liu et al., 2017). In addition, the paucity of SSR marker polymorphism in cotton resulted in insufficient linkage between markers and priority traits, which hindered the “pyramiding” of favorable QTL through MAS and fine mapping or map-based cloning.

Single-nucleotide polymorphism (SNP) markers with genome wide abundance characterized by high-throughput genotyping fulfill the need for saturated genetic map construction and are suitable for application to QTL dissection and MAS. With the release of genome sequences for cotton species such as *G. raimondii* (Paterson et al., 2012; Wang et al., 2012), *G. arboreum* (Li et al., 2014), *G. hirsutum* (Li et al., 2015; Zhang et al., 2015), and *G. barbadense* (Liu X. et al., 2015; Yuan et al., 2015), “next generation sequencing (NGS)” has been exploited and applied to construct high-density genetic maps and identify QTL in allotetraploid cotton, including restriction-site associated DNA (RAD) (Wang H. et al., 2015; Jia et al., 2016), genotyping by sequencing (GBS) (Qi et al., 2017) and specific locus amplified fragment sequencing (SLAF-seq) (Zhang et al., 2016). In addition, a SNP array for hybridization, CottonSNP63K array, has been developed (Hulse-Kemp et al., 2015) and utilized to evaluate genetic diversity, conduct genome-wide association studies (GWAS) (Hinze et al., 2017; Huang et al., 2017; Sun et al., 2017), construct genetic maps (Hulse-Kemp et al., 2015) and dissect QTL (Li C. et al., 2016; Palanga et al., 2017; Zhang Z. et al., 2017). However, the CottonSNP80K was developed based on SNP markers selected from the re-sequencing of 100 cotton cultivars, and has been rarely applied in practical breeding (Cai et al., 2017). Moreover, few of these studies were conducted for fiber quality genetic dissection, which was far removed from map-based cloning, elaborating the molecular mechanism of fiber development and further application of MAS in breeding programs. Therefore, it is urgent to identify more novel and stable QTL and develop functional markers using high-throughput genotyping as reported in rice and wheat (Wang et al., 2011; Zhang P. et al., 2017).

In the present study, the CottonSNP80K array (Cai et al., 2017) was implemented to genotype 180 F_2:8_ recombinant inbred lines (RILs) derived from the cross between a local high fiber quality cultivar, Yumian 1 and Acala Maxxa from American. Our objectives were to construct a high-density intraspecific genetic map of Upland cotton, then identify novel and stable fiber quality QTL for breeding programs and map-based cloning.

## Materials and methods

### Mapping population and statistical analysis

*Gossypium hirsutum* cultivars Yumian 1 and Acala Maxxa were crossed to produce a segregating population at Southwest University, Chongqing, China, in the summer of 2010 (Shao et al., 2014). Yumian 1 is a high fiber quality cultivar bred by a multiple-cultivar intermating program at Southwest University, Chongqing, China (Zhang et al., 2009). Acala Maxxa, with high productivity, was introduced from America (Shao et al., 2014). The F_1_ seeds were planted in Hainan, China, in the winter of 2010. A total of 180 F_2_ plants were randomly selected and self-mated at Southwest University, Chongqing, China, in the summer of 2011. Single-seed descent was conducted from the F_2:3_ generation to the F_2:8_ generation to complete a RIL population in the summer of 2015. The 180 RILs were planted in single-row plots with 0.8 m width and 5 m long at Chongqing, China, in the summer of 2016, and at Hainan, China, in the winter of 2016, respectively.

All naturally-opened bolls of the RILs and parents in three different environments were harvested for fiber quality evaluation using the HVI900 instruments (Uster®Hvispectrum, Spinlab, USA) at the Supervision Inspection and Testing Cotton Quality Center, Anyang, China. Data including fiber length (FL, mm), fiber strength (FS, cN/tex), fiber elongation (FE, %), fiber micronaire (FM), and fiber length uniformity (FU, %) were analyzed by SPSS 20.0 (SPSS, Chicago, IL, USA) for correlations, and Microsoft Excel 2013 for variance analysis (ANOVA).

### DNA extraction and genotyping

Genomic DNA was extracted from young leaves of the mapping population and parents using a modified CTAB method (Zhang et al., 2005). DNA was hybridized to CottonSNP80K arrays according to the Illumina protocols (Illumina Inc., San Diego, USA). The Illumina iScan was used to scan arrays. The GenomeStudio Genotyping software (V2011.1, Illumina, Inc.) was applied to cluster the raw data for SNP alleles and genotypes (Cai et al., 2017). The raw genotyping data were filtered, retaining only markers that behave co-dominantly and with < 40% missing data (Hulse-Kemp et al., 2015). Subsequently, the genotyping data were transformed into mapping data format (“ABH”).

### Genetic map construction

The retained SNP markers were initially partitioned and located to the 26 chromosomes based on the physical map of the *G. hirsutum* TM-1 genome sequence (Zhang et al., 2015). Then, the SNP markers on each chromosome were manually curated to identify and remove problematic markers that caused elevated numbers of double crossovers and expansions in the length of the linkage group (Hulse-Kemp et al., 2015). Ultimately, the genetic map was constructed using JoinMap 4.0 (Van Ooijen, 2006) with the default settings and with the log of odds (LOD) score of from 3 to 10 on each chromosome. Map distances were calculated using Kosambi's mapping function (Kosambi, 1944).

Chi-squared tests were employed to test loci that deviated from the 1:1 expected segregation ratio (*p* < 0.05). Regions containing at least three adjacent deviated loci were defined as segregation distortion regions (SDR) (Yu et al., 2011). Circos-0.66 (Krzywinski et al., 2009) was used to compare the colinearity of SNPs between genetic and physical maps.

### QTL mapping

MapQTL 6.0 (Van Ooijen, 2009) was employed to detect QTL by Multiple QTL mapping. A threshold of log of odds (LOD) ≥ 2.0 was used to declare suggestive QTL, as suggested (Lander and Kruglyak, 1995). Positive additive effects indicated that alleles originating from Yumian 1 increased the phenotypic value, while negative additive effects indicated that alleles derived from Acala Maxxa increased the phenotypic value. QTL with partially or fully overlapping confidence intervals was regarded as one QTL. The QTL nomenclature was designated beginning with a letter “q”, followed by the trait abbreviation as mentioned above, the chromosome number and the QTL serial number. MapChart 2.3 (Voorrips, 2002) was used to draw graphic representation of the genetic map and QTL bars representing 1-LOD likelihood intervals.

## Results

### Fiber quality evaluation

Descriptive statistics for fiber quality traits of the mapping parents and the RIL population across three environments are shown in Table 1. Acala Maxxa showed a little higher fiber length and much lower fiber micronaire than Yumian 1, however, it is indistinguishable for fiber uniformity and strength across three environments. For the RIL population, all traits showed a broad and continuous range of variation with transgressive segregation, consistent with normal distributions.

**Table 1:** Variation of fiber quality traits for Yumian 1, Acala Maxxa, and their RIL population.

**Trait^a^**	**Env^b^**	**Parents**			**Population**							
		**P_1_^c^**	**P_2_^d^**	**P_1_−P_2_**	**Max**	**Min**	**Range**	**Average**	**SD**	**Variance**	**Kurtosis**	**Skewness**
FL	2015CQ	29.25	30.10	−0.80	32.20	26.45	5.75	29.74	1.18	1.39	−0.26	−0.17
	2016CQ	29.10	30.30	−1.20	32.80	27.30	5.50	30.30	1.29	1.68	−0.78	0.10
	2016HN	29.40	29.90	−0.50	32.10	25.20	6.90	29.21	1.42	2.03	−0.05	−0.49
FU	2015CQ	84.85	84.20	0.70	87.60	83.05	4.55	85.15	0.98	0.97	−0.25	0.11
	2016CQ	84.80	87.00	−2.20	88.80	83.90	4.90	86.26	1.09	1.18	−0.32	0.14
	2016HN	84.90	81.40	3.50	87.60	80.60	7.00	84.02	1.42	2.01	−0.09	−0.07
FS	2015CQ	32.85	32.55	0.30	37.90	27.80	10.10	32.51	2.21	4.89	−0.13	0.36
	2016CQ	35.80	37.40	−1.60	45.40	29.90	15.50	35.95	3.20	10.25	0.41	0.61
	2016HN	29.90	27.70	2.20	33.50	23.40	10.10	29.13	1.92	3.67	0.17	0.12
FM	2015CQ	4.10	3.40	0.70	4.95	3.30	1.65	3.99	0.34	0.12	−0.26	0.18
	2016CQ	4.80	4.20	0.60	5.50	3.40	2.10	4.40	0.43	0.19	0.03	−0.01
	2016HN	3.40	2.60	0.80	4.40	2.60	1.80	3.57	0.39	0.15	−0.33	0.07
FE	2015CQ	6.75	6.70	0.10	6.90	6.45	0.45	6.69	0.09	0.01	0.01	−0.16
	2016CQ	6.80	6.80	0.00	7.00	6.50	0.50	6.76	0.13	0.02	−0.64	0.03
	2016HN	6.70	6.60	0.10	6.80	6.40	0.40	6.62	0.08	0.01	−0.15	−0.30

a *FL, Fiber length; FU, fiber uniformity; FS, fibers strength; FM, fiber micronaire; FE, fiber elongation*.

b *2015CQ, 2015 in Chongqing; 2016CQ, 2016 in Chongqing; 2016HN, 2016 in Hainan*.

c *P_1_, Yumian 1*.

d *P_2_, Acala Maxxa*.

Correlation analysis across three environments was conducted separately (Table 2). Results of three environments showed that FL had significant positive correlations with FU, FS, and FE; FS had significant positive correlations with FU and FE; and FE had significant positive correlations with FU, respectively.

**Table 2:** Correlation analysis among fiber quality traits across three environments.

**Env^a^**	**Traits^b^**	**FL**	**FU**	**FS**	**FM**	**FE**
2015CQ	FL	1				
	FU	0.368^**^	1			
	FS	0.667^**^	0.389^**^	1		
	FM	−0.377	−0.045	−0.259	1	
	FE	0.796^**^	0.403^**^	0.798^**^	−0.116	1
2016CQ	FL	1				
	FU	0.323^**^	1			
	FS	0.660^**^	0.454^**^	1		
	FM	−0.282	0.018	−0.339	1	
	FE	0.807^**^	0.442^**^	0.795^**^	0.01	1
2016HN	FL	1				
	FU	0.432^**^	1			
	FS	0.600^**^	0.329^**^	1		
	FM	−0.261	0.067	−0.007	1	
	FE	0.747^**^	0.396^**^	0.698^**^	−0.096	1

** *Indicate significance at the 0.01 level, respectively*.

a *FL, Fiber length; FU, fiber uniformity; FM, fiber micronaire; FE, fiber elongation; FS, fiber strength*.

b *2015CQ, 2015 in Chongqing; 2016CQ, 2016 in Chongqing; 2016HN, 2016 in Hainan*.

Furthermore, analyses of variance indicated highly significant genotypic and environmental effects (*P* < 0.01) for all tested fiber quality traits (Table 3), which suggested that environmental factors played a crucial role in the development of fiber quality traits.

**Table 3:** Analysis of variance (ANOVA) for fiber quality traits across three environments for the Yumian 1 × Acala Maxxa RIL population.

**Trait^a^**	**Factor**	**Sum of squares**	**DF^b^**	**MS**	**F**
FL	Environment	107.97	2	53.99	110.67^**^
	Genotype	699.12	169	4.14	8.48^**^
	Error	164.87	338	0.49	
FU	Environment	416.25	2	208.13	312.74^**^
	Genotype	486.63	169	2.88	4.32^**^
	Error	224.94	338	0.67	
FS	Environment	4, 023.42	2	2, 011.71	1004.94^**^
	Genotype	2, 462.67	169	14.57	7.27^**^
	Error	676.62	338	2.00	
FM	Environment	57.81	2	28.91	520.29^**^
	Genotype	58.93	169	0.35	6.27^**^
	Error	18.78	338	0.06	
FE	Environment	1.87	2	0.93	240.54^**^
	Genotype	3.69	169	0.02	5.62^**^
	Error	1.31	338	0.00	

** *Indicates significance at the 0.01 level*.

a *FL, Fiber length; FU, fiber uniformity; FM, fiber micronaire; FE, fiber elongation; FS, fiber strength*.

b *Excluded missing data when analysis the variance*.

### Map construction

CottonSNP80K arrays were used for screening 180 RILs, with 12,318 SNP (15.84%) polymorphic between the two mapping parents and segregating in the population, of which 12,116 were mapped on 26 chromosomes, accounting for 15.58% of the total of 77,774 SNP markers. The final genetic map spanned 3,741.81 cM with an average distance of 0.31 cM between consecutive loci (Table 4; Figure S1). The At subgenome included 5,975 SNP markers and covered 1,979.72 cM with an average of 0.33 cM between consecutive loci, while the Dt subgenome contained 6141 SNP markers and spanned 1,762.09 cM with an average of 0.29 cM between consecutive loci. Uneven distribution of SNP markers was observed. Chr17 had the most loci (707), while Chr02 had the least (197). The average length of the 26 chromosomes was 143.91 cM. The longest chromosome was Chr05 (209.49), and the shortest was Chr25 (102.04). Nineteen gaps (>15 cM) were present in this genetic map, including 10 on the At subgenome and 9 on the Dt subgenome (Table 4).

**Table 4:** Summary of the SNP genetic map of the Yumian 1 × Acala Maxxa RIL population.

**Chromosome**	**No. SNP**	**Length (cM)**	**Average density**	**Gaps > 15 cM**	**Distorted markers**	**Distortion rate (%)**	**SDR^a^**
Chr01	371	154.59	0.42	1	175	47.17	2
Chr02	197	105.23	0.53	1	8	4.06	1
Chr03	686	188.74	0.28	0	83	12.10	2
Chr04	250	120.28	0.48	3	8	3.20	1
Chr05	640	209.49	0.33	1	158	24.69	2
Chr06	207	121.96	0.59	2	53	25.60	2
Chr07	672	166.06	0.25	0	143	21.28	1
Chr08	615	144.14	0.23	1	145	23.58	3
Chr09	486	155.48	0.32	0	483	99.38	1
Chr10	524	159.29	0.30	0	110	20.99	2
Chr11	345	178.19	0.52	0	123	35.65	5
Chr12	509	150.39	0.30	0	121	23.77	1
Chr13	473	125.87	0.27	1	124	26.22	2
At subgenome	5,975	1, 979.72	0.33	10	1,734	29.02	25
Chr14	604	172.51	0.29	1	229	37.91	1
Chr15	453	136.57	0.30	1	48	10.60	1
Chr16	315	138.25	0.44	2	62	19.68	2
Chr17	707	119.58	0.17	0	77	10.89	2
Chr18	604	125.65	0.21	1	180	29.80	2
Chr19	533	168.37	0.32	1	271	50.84	3
Chr20	369	126.77	0.34	0	209	56.64	1
Chr21	501	157.36	0.31	2	459	91.62	1
Chr22	373	114.02	0.31	0	103	27.61	2
Chr23	376	131.68	0.35	0	57	15.16	2
Chr24	454	122.30	0.27	1	85	18.72	2
Chr25	340	102.04	0.30	0	168	49.41	1
Chr26	512	147.00	0.29	0	295	57.62	4
Dt subgenome	6,141	1, 762.09	0.29	9	2,243	36.52	24
Total	12,116	3, 741.81	0.31	19	3,977	32.82	49

a *SDR, Segregation distortion region*.

### Segregation distortion

Among the total of 12,116 mapped SNPs markers, 32.82% (3977) showed segregation distortion (*P* < 0.05) with most (3743) enriched for Yumian 1 alleles (Table 4; Figure S1). These segregation distorted markers (SDMs) were unevenly distributed on the 26 chromosomes (1734 on At subgenome and 2243 on Dt subgenome). Chr09 and Chr21 contained the most SDMs (483 and 459, respectively) with the highest percentage of 99.38 and 91.62%, respectively; Chr02 and Chr04 included the fewest SDMs (8 and 8, respectively) with the percentage lower than 5%. The SDMs were aggregated into 49 segregation distortion regions (SDRs) on 26 chromosomes, including 25 on At and 24 on Dt chromosomes. Notably, SDMs located on six SDRs (SDR02-1, SDR05-1, SDR08-2, SDR13-2, SDR15-1, SDR26-4) skewed toward the paternal (Acala Maxxa) alleles, and others skewed toward the maternal (Yumian 1) alleles (Figure S1).

### Colinearity analysis

**Figure 1:**
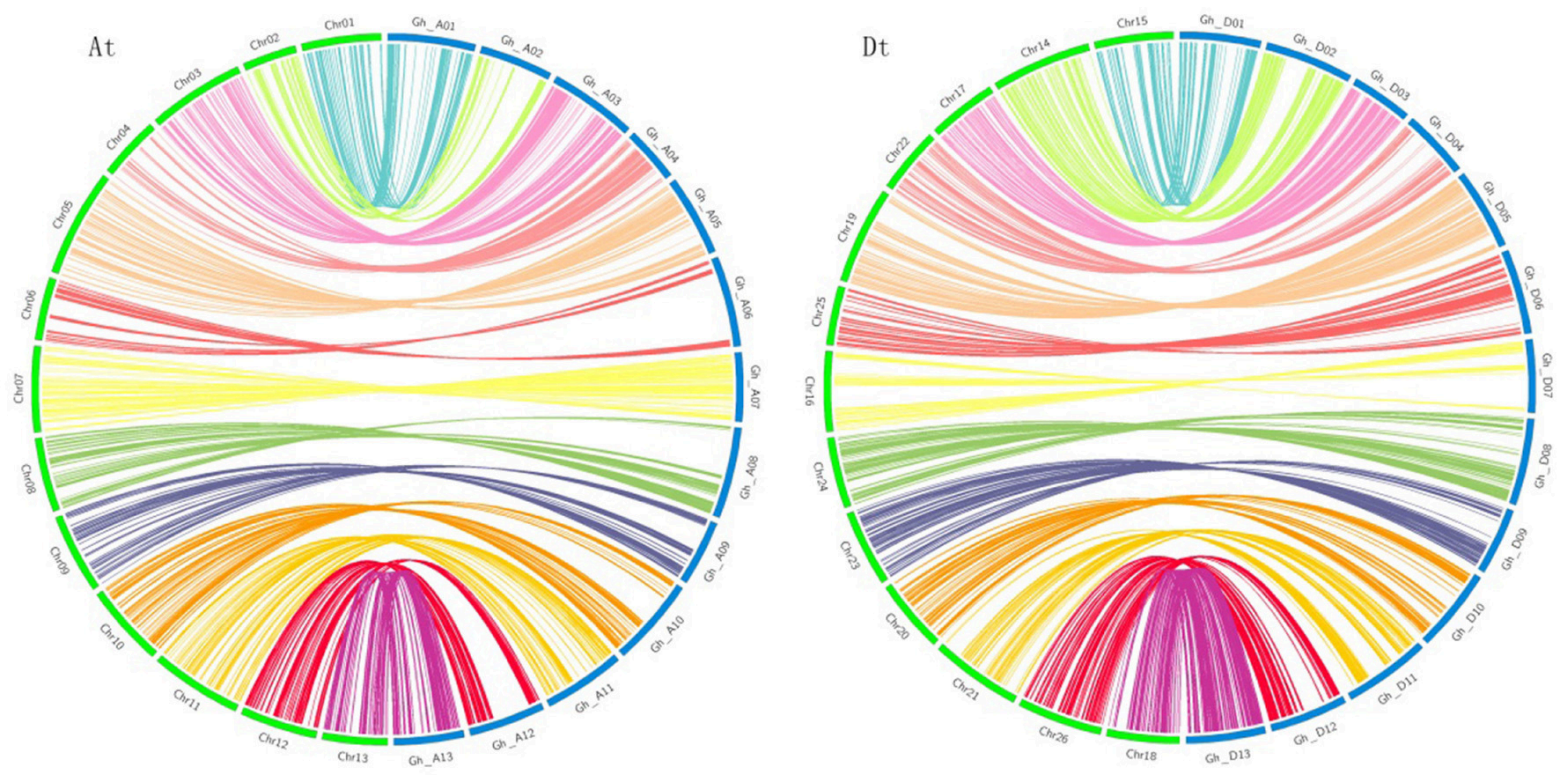
Colinearity between genetic map (green) and physical map (blue). (At) Colinearity for At subgenome. (Dt) Colinearity for Dt subgenome.

To validate the accuracy and precision of the genetic map, we compared it with the physical maps of the *G. hirsutum* reference genome sequence (Zhang et al., 2015) (Figure 1). The vast majority of loci on the genetic map were reasonably consistent with their corresponding physical locations, which suggested the high quality of the genetic map. However, several deviations on Chr02 and Chr07 were observed. In total, 3,741.81 cM of the genetic map corresponded to 1,934.65 Mb of the physical map, spanning 97.99% of the genome length of the 26 chromosomes. All chromosomes showed good coverage of the physical map except for Chr21 with 89.33% coverage (Table S1).

### QTL mapping of fiber quality traits

A total of 104 QTL controlling fiber quality traits were detected in this study (Table S2; Figure S1), explaining 5.0–18.8% of the total phenotypic variance with LOD values ranging from 2.0 to 7.7. These QTL were unevenly distributed on 22 chromosomes except for Chr03, Chr04, Chr06, and Chr12, including 51 QTL on the At subgenome and 53 on the Dt subgenome. Yumian 1 contributed positive additive effects at 48 QTL to increase relevant trait values, whereas 56 QTL alleles with negative additive effects derived from Acala Maxxa. Significantly, 35 QTL were detected in 2 environments (Table S2) and 25 in three environments (Table 5).

**Table 5:** Stable QTL for fiber quality traits identified across three environments in the Yumian 1 × Acala Maxxa RIL population.

**Trait^a^**	**QTL**	**Environment^b^**	**Flanking markers**	**Nearest locus**	**Location**	**LOD**	**Additive effect^c^**	**PVE^d^ (%)**
FL	qFL01.1	2015CQ	TM1432-TM3147	TM1620	94.854	3.38	0.38	8.7
		2016CQ	TM1162-TM3147	TM1620	94.854	4.34	0.47	11.1
		2016HN	TM1140-TM1470	TM1162	90.402	2.02	0.34	5.0
	qFL08.1	2015CQ	TM21793-TM21830	TM21807	8.857	6.32	0.50	15.7
		2016CQ	TM21777-TM21814	TM21805	7.398	7.16	0.58	17.6
		2016HN	TM21805-TM21923	TM21814	10.316	4.84	0.53	11.7
	qFL15.1	2015CQ	TM48765-TM49556	TM49428	91.518	2.54	−0.33	6.7
		2016CQ	TM48872-TM49556	TM49428	91.518	2.58	−0.36	6.7
		2016HN	TM48600-TM49428	TM48737	73.361	2.48	−0.38	6.2
	qFL20.1	2015CQ	TM74863-TM75013	TM74991	94.431	3.56	−0.39	9.2
		2016CQ	TM74977-TM75013	TM74991	94.431	2.12	−0.33	5.6
		2016HN	TM74758-TM74985	TM74955	89.112	5.72	−0.58	13.6
	qFL21.2	2015CQ	TM76374-TM76405	TM76404	109.189	3.54	−0.42	9.1
		2016CQ	TM76374-TM76405	TM76404	109.189	4.00	−0.49	10.3
		2016HN	TM76378-TM76405	TM76404	109.189	2.04	−0.37	5.1
	qFL23.1	2015CQ	TM72938-TM72973	TM72959	130.821	4.48	0.43	11.4
		2016CQ	TM72952-TM72973	TM72971	131.681	3.22	0.41	8.4
		2016HN	TM72885-TM72973	TM72952	126.570	4.42	0.51	10.7
	qFL24.1	2015CQ	TM68725-TM69012	TM68991	62.109	4.56	0.45	11.6
		2016CQ	TM68643-TM69000	TM68932	61.540	3.14	0.41	8.1
		2016HN	TM68725-TM69014	TM68991	62.109	3.20	0.45	7.9
FU	qFU05.1	2015CQ	TM13164-TM13321	TM13220	171.894	6.12	−0.42	15.3
		2016CQ	TM13164-TM13347	TM13329	177.699	6.70	−0.48	16.6
		2016HN	TM13144-TM13325	TM13220	171.894	2.44	−0.38	6.0
	qFU15.3	2015CQ	TM50014-TM50078	TM50051	109.549	4.46	−0.36	11.4
		2016CQ	TM50014-TM50078	TM50051	109.549	2.38	−0.30	6.3
		2016HN	TM49771-TM50078	TM50051	109.549	2.70	−0.39	6.7
	qFU16.1	2015CQ	TM63183-TM63269	TM63214	13.639	4.44	−0.37	11.3
		2016CQ	TM63183-TM63217	TM63187	11.080	3.74	−0.38	9.6
		2016HN	TM63237-TM63411	TM63318	22.338	2.72	−0.40	6.7
	qFU20.1	2015CQ	TM74740-TM75048	TM74991	94.431	6.06	−0.42	15.1
		2016CQ	TM74755-TM75033	TM74991	94.431	4.32	−0.39	11.0
		2016HN	TM74755-TM74903	TM74834	85.390	3.28	−0.45	8.1
FS	qFS01.1	2015CQ	TM1140-TM3147	TM1162	90.402	3.42	0.70	8.8
		2016CQ	TM1613-TM3147	TM2506	99.829	3.10	1.01	8.1
		2016HN	TM1140-TM1432	TM1162	90.402	4.12	0.65	10.0
	qFS08.1	2015CQ	TM21777-TM21825	TM21793	4.996	3.78	0.76	9.7
		2016CQ	TM21777-TM21814	TM21793	4.996	3.12	1.00	8.1
		2016HN	TM21777-TM21923	TM21805	7.398	2.80	0.54	6.9
	qFS13.4	2015CQ	TM47688-TM47730	TM47705	121.794	5.40	−0.91	13.6
		2016CQ	TM47699-TM47716	TM47705	121.794	3.30	−1.04	8.5
		2016HN	TM47694-TM47730	TM47705	121.794	5.90	−0.79	14.0
	qFS15.2	2015CQ	TM48872-TM49692	TM49428	91.518	4.44	−0.80	11.3
		2016CQ	TM48872-TM49695	TM49428	91.518	3.90	−1.09	10.0
		2016HN	TM48872-TM49518	TM49428	91.518	2.82	−0.54	7.0
	qFS15.3	2015CQ	TM50014-TM50078	TM50051	109.549	2.00	−0.55	5.3
		2016CQ	TM50014-TM50078	TM50062	109.549	2.10	−0.82	5.5
		2016HN	TM49556-TM49695	TM49562	98.749	3.32	−0.58	8.1
	qFS19.1	2015CQ	TM57119-TM57276	TM57229	50.938	3.08	0.73	8.0
		2016CQ	TM57193-TM57264	TM57229	50.938	2.34	0.93	6.1
		2016HN	TM57143-TM57294	TM57167	46.474	3.22	0.63	7.9
	qFS20.1	2015CQ	TM74946-TM75012	TM74991	94.431	3.86	−0.76	9.9
		2016CQ	TM74977-TM75018	TM74991	94.431	2.74	−0.93	7.2
		2016HN	TM74834-TM75011	TM74955	89.112	4.74	−0.71	11.4
FM	qFM16.2	2015CQ	TM63214-TM63269	TM63238	15.361	4.94	0.13	12.5
		2016CQ	TM63332-TM63411	TM63377	27.434	4.60	0.16	11.7
		2016HN	TM63214-TM63269	TM63238	15.361	2.68	0.11	6.7
	qFM24.1	2015CQ	TM69870-TM69911	TM69890	119.333	5.34	−0.13	13.5
		2016CQ	TM69856-TM69911	TM69890	119.333	4.24	−0.15	10.9
		2016HN	TM69873-TM69913	TM69890	119.333	3.20	−0.12	7.9
FE	qFE01.1	2015CQ	TM1140-TM2506	TM1162	90.402	4.66	0.03	11.8
		2016CQ	TM1140-TM3147	TM1162	90.402	3.06	0.04	8.0
		2016HN	TM1140-TM1432	TM1162	90.402	4.34	0.03	10.5
	qFE08.1	2015CQ	TM21777-TM21818	TM21793	4.996	6.68	0.04	16.5
		2016CQ	TM21777-TM21814	TM21793	4.996	7.70	0.06	18.8
		2016HN	TM21793-TM21923	TM21814	10.316	3.50	0.03	8.6
	qFE15.2	2015CQ	TM48872-TM49557	TM49428	91.518	5.48	−0.03	13.8
		2016CQ	TM48872-TM49533	TM49428	91.518	3.24	−0.04	8.4
		2016HN	TM48872-TM49557	TM49534	96.740	4.50	−0.03	10.9
	qFE20.1	2015CQ	TM74946-TM75011	TM74991	94.431	4.48	−0.03	11.4
		2016CQ	TM74977-TM75012	TM74991	94.431	3.70	−0.04	9.6
		2016HN	TM74863-TM74985	TM74955	89.112	5.66	−0.03	13.5
	qFE24.1	2015CQ	TM68898-TM69014	TM68991	62.109	4.88	0.03	12.4
		2016CQ	TM68802-TM69013	TM68991	62.109	2.18	0.03	5.7
		2016HN	TM68746-TM69013	TM68991	62.109	4.30	0.03	10.4

a *FL, Fiber length; FU, fiber uniformity; FS, fiber strength; FE, fiber elongation; FM, fiber micronaire*.

b *2015CQ: 2015 in Chongqing; 2016CQ: 2016 in Chongqing; 2016HN: 2016 in Hainan*.

c *Positive additive effects indicated that Yumian 1 alleles increased the phenotypic value, negative additive effects suggested that Acala Maxxa alleles increased the phenotypic value*.

d *Phenotypic variance explained*.

### Fiber length

Twenty-one QTL controlling fiber length were detected on 17 chromosomes (with two each on Chr05, Chr10, Chr11, and Chr21), explaining 5.0–17.6% of phenotypic variance (Table S2; Figure S1). Among these QTL, 11 favorable alleles that increased fiber length were derived from Yumian 1, whereas 10 were contributed by Acala Maxxa. Seven QTL (qFL01.1, qFL08.1, qFL15.1, qFL20.1, qFL21.1, qFL23.1, and qFL24.1) were detected across three environments, with six explaining ~10% of phenotypic variance (Table 5).

### Fiber uniformity

Sixteen QTL were identified on 11 chromosomes and explained 5.5–17.8% of the phenotypic variance. Chr14, Chr15, Chr21, and Chr24 included 2, 3, 2, and 2 QTL, respectively (Table S2; Figure S1). There were only four QTL for which alleles increasing fiber uniformity came from Yumian 1, whereas 12 favorable QTL alleles originated from Acala Maxxa. Furthermore, four QTL (qFU05.1, qFU15.3, qFU16.1, and qFU20.1), which explained more than 10% phenotypic variance were stable and detected in three environments (Table 5).

### Fiber strength

Twenty-seven QTL were detected on 18 chromosomes and explained 5.1–14.0% of the phenotypic variance. On Chr09, Chr13, Chr15, Chr16, and Chr19, we identified 3, 4, 3, 2, and 2 QTL, respectively (Table S2; Figure S1). Favorable alleles of 11 QTL which increased fiber strength were derived from Yumian 1, whereas others were contributed by Acala Maxxa. Notably, there were seven stable QTL (qFS01.1, qFS08.1, qFS13.4, qFS15.2, qFS15.3, qFS19.1, and qFS20.1) identified across all environments. Moreover, qFS13.4, qFS15.2, and qFS20.1 explained ~10% of the phenotypic variance (Table 5).

### Fiber micronaire

Eighteen QTL were identified on 14 chromosomes, explaining 5.2–17.2% of the phenotypic variance. And there were 2, 3, and 2 QTL detected on Chr08, Chr13, and Chr16, respectively (Table S2; Figure S1). Among these QTL, six favorable alleles for decreasing fiber micronaire value were contributed by Yumian 1, the rest came from Acala Maxxa. There were only two stable QTL (qFM16.2 and qFM24.1) identified in three environments. However, five QTL (qFM09.1, qFM13.2, qFM16.1, qFM19.1, and qFM25.1) also explained ~10% of phenotypic variance (Table S2).

### Fiber elongation

Twenty-two QTL were mapped on 15 chromosomes, explaining 5.2–18.8% of the phenotypic variance. Chr10, Chr11, Chr13, Chr15, and Chr18 contained 3, 2, 3, 2, and 2 QTL on different regions, respectively (Table S2; Figure S1). Among these QTL, 10 favorable alleles increasing the trait value came from Yumian 1, whereas the rest were derived from Acala Maxxa. There were five stable QTL (qFE01.1, qFE08.1, qFE15.2, qFE20.1, and qFE24.1) detected in three environments (Table 5).

### QTL cluster analysis

QTL clusters were defined as regions which contained multiple QTL associated with various traits within ~20 cM (Said et al., 2015b). In this study, there were 19 QTL clusters on16 chromosomes (Table 6 and Table S3). Significantly, both Chr01-cluster-1 and Chr08-cluster-1 contained three stable QTL for FL, FS, and FE, explaining ~10% of the phenotypic variance. Chr05 carried 2 clusters, both of which included three QTL, with a stable QTL (qFU05.1) mapped on Chr05-cluster-2. There were also two clusters on Chr13—Chr13-cluster-1 included 7 QTL, qFE13.1, qFM13.1, and qFS13.1 mapped on almost the same location with positive additive effects, and another four QTL with negative additive effects overlapping these; Chr13-cluster-2 contained four QTL including a stable QTL, qFS13.4, identified across three environments. Among two clusters on Chr15, Chr15-cluster-1 conferred 3 QTL identified only in 2016HN and Chr15-cluster-2 carried 6 QTL which included five stable QTL identified in three environments. Chr16-cluster-1 included 6 QTL with two stable QTL (qFU16.1 and qFM16.2), and two sets of QTL for FS and FM were identified in obviously different regions. Chr20-cluster-1 contained 4 stable QTL except for FM, explaining about 10% of the phenotypic variance. Chr23-cluster-1 carried 3 QTL including a stable QTL, qFL23.1. Chr24-cluster-1 contained 4 QTL, with 2 stable QTL, qFE24.1 and qFL24.1. Details were summarized in Table S3. In partial summary, there were 9 QTL clusters which carried at least one stable QTL—in addition, stable QTL qFS19.1, qFL21.2, and qFM24.1 each linked to another QTL should also be priorities for further study and deployment.

**Table 6:** QTL Clusters for fiber quality traits identified across three environments in the Yumian 1 × Acala Maxxa RIL population.

**Cluster**	**QTL^a^**	**Flanking markers^b^**
Chr01-cluster-1	qFL01.1^#^, qFS01.1^#^, qFE01.1^#^	TM1140-TM3147
Chr02-cluster-1	qFL02.1, qFS02.1, qFE02.1	TM4519-TM4763
Chr05-cluster-1	qFL05.1, qFS05.1, qFE05.1	TM10778-TM10916
Chr05-cluster-2	qFL05.2, qFU05.1^#^, qFM05.1	TM12925-TM13329
Chr07-cluster-1	qFS07.1, qFM07.1, qFE07.1	TM20539-TM21347
Chr08-cluster-1	qFL08.1^#^, qFS08.1^#^, qFE08.1^#^	TM21777-TM21923
Chr09-cluster-1	qFL09.1, qFS09.2, qFM09.1	TM33017-TM33444
Chr11-cluster-1	qFL11.2, qFS11.1, qFM11.1, qFE11.2	TM39464-TM39697
Chr13-cluster-1	qFS13.1, qFS13.2, qFS13.3, qFM13.1, qFM13.2, qFE13.1, qFE13.2	TM43304-TM43875
Chr13-cluster-2	qFU13.1, qFS13.4^#^, qFM13.3, qFE13.3	TM47648-TM47738
Chr15-cluster-1	qFU15.1, qFS15.1, qFE15.1	TM48070-TM48361
Chr15-cluster-2	qFL15.1^#^, qFU15.2, qFU15.3^#^, qFS15.2^#^, qFS15.3^#^, qFE15.2^#^	TM48600-TM50078
Chr16-cluster-1	qFL16.1, qFU16.1, qFS16.1^#^, qFS16.2, qFM16.1, qFM16.2^#^	TM63172-TM63428
Chr18-cluster-1	qFL18.1, qFS18.1, qFE18.1, qFE18.2	TM80134-TM80469
Chr20-cluster-1	qFL20.1^#^, qFU20.1^#^, qFS20.1^#^, qFE20.1^#^	TM74740-TM75048
Chr21-cluster-1	qFL21.1, qFS21.1, qFM21.1, qFE21.1	TM75301-TM75569
Chr23-cluster-1	qFL23.1^#^, qFM23.1, qFE23.1	TM72885-TM72973
Chr24-cluster-1	qFL24.1^#^, qFU24.1, qFS24.1, qFE24.1^#^	TM68643-TM69014
Chr26-cluster-1	qFL26.1, qFS26.1, qFE26.1	TM79192-TM79332

# *Indicated stable QTL identified across three environments*.

a *FL, Fiber length; FU, fiber uniformity; FS, fiber strength; FE, fiber elongation; FM, fiber micronaire*.

b *Flanking Markers contained a larger region consisting all QTL in a giving cluster*.

## Discussion

### High-density genetic map construction

High-throughput genotyping methods are useful to evaluate genetic diversity, construct genetic maps, and dissect the genetic architecture of important traits (Truco et al., 2013). Previously, the narrow genetic base and two closely related subgenomes of Upland cotton had hindered the identification of SNP markers (Islam et al., 2015). With the release of genome sequences of several cotton species (Paterson et al., 2012; Wang et al., 2012; Li et al., 2014, 2015; Liu X. et al., 2015; Yuan et al., 2015; Zhang et al., 2015) and the improvement of *in silico* methods, CottonSNP63K and CottonSNP80K arrays were develop and applied to cotton research (Hulse-Kemp et al., 2015; Cai et al., 2017).

Here, CottonSNP80K arrays were employed to genotype 180 RILs and construct a high density genetic map containing 12116 SNP markers which spanned a total recombinational length of 3741.81 cM with an average distance of 0.31 cM between consecutive markers. To our knowledge, this is the first application for genetic map construction of the CottonSNP80K array, which is based on SNP markers selected from the re-sequencing of 100 cotton cultivars (Cai et al., 2017). Compared with previous intraspecific SNP genetic maps (Hulse-Kemp et al., 2015; Jia et al., 2016; Zhang et al., 2016; Qi et al., 2017), the present map was efficient and possessed more SNP markers, due to both the high-efficiency genotyping of the CottonSNP80K array (Cai et al., 2017) and the use of relatively divergent parents, specifically the Chinese cultivar Yumian 1 and the American cultivar Acala Maxxa.

Good colinearity between the present genetic map and the corresponding physical map suggests the accuracy and precision of the map. Colinearity analysis also indicated that the present genetic map had good coverage of the cotton genome, except for Chr21 due to few polymorphic loci in a 6.48 Mb region at the end of the chromosome. Nonetheless, the present map still harbored 19 gaps (>15 cM). Such defects were also widespread among genetic maps constructed using SSR (Liu D. et al., 2015; Li, X. et al., 2016; Liu et al., 2017) and SNP markers in cotton (Hulse-Kemp et al., 2015; Jia et al., 2016; Qi et al., 2017; Sun et al., 2017) and other crops (Shao et al., 2015; Clarke et al., 2016; Montero-Pau et al., 2017).

### Segregation distortion

Segregation distortion, recognized as a potentially powerful evolutionary force (Taylor and Ingvarsson, 2003), is widespread in mapping populations. It is more serious in RIL populations because of genetic drift (Zhang et al., 2009) associated with both natural and artificial selection for several generations, during which time lethality, partial male or female sterility, gametic selection, zygotic selection, and/or pollen spine development happen naturally (Xian-Liang et al., 2007). In the present study, the frequency of segregation distortion (32.82%) was higher than in a previous F_2_ SSR map (16.9%) with the same parents (Shao et al., 2014), which suggested that RIL development contributed to more distorted segregation once again. Furthermore, previous SNP maps constructed for RIL populations of Upland cotton showed segregation distortion ranging from 13.29 to 63.77% (Wang, Y. et al., 2015; Li C. et al., 2016; Zhang et al., 2016; Zhang Z. et al., 2017), implying that the divergence level of the mapping parents may play a critical factor in segregation distortion (Paterson et al., 1988). Curiously, most distorted loci (89.50%) skewed toward Yumian 1, whereas only 10.50% skewed toward Acala Maxxa, consistent with previous studies that 74.5–95% of the distorted loci favored Yumian 1 alleles (Hu et al., 2008; Tan et al., 2014; Tang et al., 2014; Liu D. et al., 2015; Liu et al., 2017), suggesting that specific features of its genome may favor Yumian 1 genotypes.

### QTL mapping

While fiber quality traits of the mapping parents were similar, except for fiber micronaire, significant differences among the RIL population and variance analysis showed that the parents carried different alleles for many fiber quality traits. We detected 104 QTL controlling fiber quality traits, with 42 favorable alleles from Yumian 1 and 62 from Acala Maxxa. This relatively rich QTL diversity between parents with similar phenotypes is consistent with prior studies (Shen et al., 2005; Tan et al., 2014; Li C. et al., 2016; Liu et al., 2017), supporting the ideas that different elite cultivars can have different favorable alleles for the same traits. That Acala Maxxa conferred twice as many (12 vs. 6) favorable fiber micronaire alleles as Yumian 1 was consistent with significant dissimilarity of this trait between parents, in accordance with the expectation that selection of one superior and one inferior parent will confer rich segregation for traits of interest (Shen et al., 2007; Sun et al., 2012).

Significant positive correlations among fiber quality traits was consistent with the phenomenon that most (74 of 104) QTL were concentrated in 19 QTL clusters, similar to previous studies (Zhang et al., 2009; Li C. et al., 2016; Liu et al., 2017). This suggested that certain traits involved multiple genes concentrated in certain genomic regions (Said et al., 2013) or were pleiotropic effects of single genes. Curiously, Chr13-cluster-1 and Chr18-cluster-1 included two sets of linked QTL with opposite additive effects, respectively, suggesting that favorable alleles at some loci were closely linked with unfavorable alleles at nearby loci. Identification of complementary haplotypes and use of tightly linked or functional markers to identify recombinants and pyramid favorable alleles through MAS may be a highly effective breeding strategy in such cases.

Interestingly, we found that most fiber micronaire QTL were mapped on a segregated or skewed region relative to QTL controlling others traits. For example, fine mapping of clustered QTL for fiber quality on chr07 also proved that qFM-chr.7 was tightly linked to qFL-chr.7 and qFS-chr.7, but not pleiotropic (Cao et al., 2015). The additive efforts of the fiber micronaire QTL in clusters had no constant positive or negative correlation with other traits, resulting in insignificant phenotypic correlation between fiber micronaire and the other traits in the current study. These interesting results had not previously been reported in QTL mapping using Yumian 1 as a parent (Hu et al., 2008; Zhang et al., 2009; Tan et al., 2014; Tang et al., 2014; Liu et al., 2017).

### Stable and common QTL

Variance analysis suggested that environmental effects play an important role in the development of fiber quality traits, with only 25 QTL identified in all three test environments. These stable QTL, especially those distributed into clusters, were credible and deserved priority for fine mapping and identification of candidate genes to elaborate molecular mechanisms of fiber development.

We compared the detected QTL with the CottonQTLdb (Release 2.2, February 01, 2017) (Said et al., 2013, 2015a,b) through the physical position of the nearest marker(s). In total, there were 10 QTL clusters (Table S4) that shared almost the same physical position of the TM-1 reference genome (Zhang et al., 2015), while stable QTL or clusters of qFS19.1, qFL21.2, qFM24.1, Chr08-cluster-1 and Chr20-cluster-1 were newly found in this study. These common QTL clusters (Table S4) and novel stable QTL or clusters (Tables 5, 6) would be priorities for implementation in breeding programs using MAS to improve cotton fiber quality traits. In contrast, unstable and uncommon QTL may be attributed to imprecise coarse mapping that caused inconsistencies among different genetic backgrounds (Said et al., 2015b) or may be specific to particular environments or genotypes.

## Conclusion

In this study, a high-density genetic map containing 12116 SNP markers was constructed, which spanned 3741.81 cM of recombinational length with an average distance of 0.31 cM between markers. Twenty-two stable QTL were distributed into 9 QTL clusters, and three additional stable QTL (QTL qFS19.1, qFL21.2, and qFM24.1) were credible and deserved priority for fine mapping to identify candidate genes and be deployed using MAS in cotton breeding programs.

## Author contributions

ZheZ designed and supervised the experiments and contributed final editing of manuscript; ZhaT performed the experiments and drafted the manuscript; ZhaT and ZhiZ analyzed the data of map and QTL; ZhaT, XS, and QL performed phenotyping data analysis; YS, PY, WW, XL, and CC participated in field trials and DNA extraction; DeL, KG, JZ, ZhoT, and DaL reviewed the manuscript. All authors have read and approved the final manuscript.

### Conflict of interest statement

The authors declare that the research was conducted in the absence of any commercial or financial relationships that could be construed as a potential conflict of interest.

## References

[B1] Cai C. Zhu G. Zhang T. Guo W. (2017). High-density 80 K SNP array is a powerful tool for genotyping *G. hirsutum* accessions and genome analysis. BMC Genomics 18:654. 10.1186/s12864-017-4062-2 28835210 PMC5569476

[B2] Cao Z. Zhu X. Chen H. Zhang T. (2015). Fine mapping of clustered quantitative trait loci for fiber quality on chromosome 7 using a *Gossypium barbadense* introgressed line. Mol. Breed. 35:215 10.1007/s11032-015-0393-3

[B3] Chen Z. J. Scheffler B. E. Dennis E. Triplett B. A. Zhang T. Guo W.. (2007). Toward sequencing cotton (*Gossypium*) genomes. Plant Physiol. 145, 1303–1310. 10.1104/pp.107.107672 18056866 PMC2151711

[B4] Clarke W. E. Higgins E. E. Plieske J. Wieseke R. Sidebottom C. Khedikar Y.. (2016). A high-density SNP genotyping array for *Brassica napus* and its ancestral diploid species based on optimised selection of single-locus markers in the allotetraploid genome. Theor. Appl. Genet. 129, 1887–1899. 10.1007/s00122-016-2746-7 27364915 PMC5025514

[B5] Hinze L. L. Hulse-Kemp A. M. Wilson I. W. Zhu Q. H. Llewellyn D. J. Taylor J. M.. (2017). Diversity analysis of cotton (*Gossypium hirsutum* L.) germplasm using the CottonSNP63K Array. BMC Plant Biol. 17:37. 10.1186/s12870-017-0981-y 28158969 PMC5291959

[B6] Hu W. J. Zhang X. Y. Zhen Z. T. Zhen G. W. (2008). Molecular tagging and source analysis of QTL for elite fiber quality in Upland Cotton. Acta Agron. Sin. 34, 578–586. 10.3724/SP.J.1006.2008.00578

[B7] Huang C. Nie X. Shen C. You C. Li W. Zhao W.. (2017). Population structure and genetic basis of the agronomic traits of upland cotton in China revealed by a genome-wide association study using high-density SNPs. Plant Biotechnol. J. 15, 1374–1386. 10.1111/pbi.12722 28301713 PMC5633765

[B8] Hulse-Kemp A. M. Lemm J. Plieske J. Ashrafi H. Buyyarapu R. Fang D. D.. (2015). Development of a 63K SNP array for cotton and high-density mapping of intraspecific and interspecific populations of *Gossypium* spp. G3 5, 1187–1209. 10.1534/g3.115.018416 25908569 PMC4478548

[B9] Islam M. S. Thyssen G. N. Jenkins J. N. Fang D. D. (2015). Detection, validation, and application of genotyping-by-sequencing based single nucleotide polymorphisms in Upland Cotton. Plant Genome 8, 1–10. 10.3835/plantgenome2014.07.0034 33228292

[B10] Jia X. Pang C. Wei H. Wang H. Ma Q. Yang J.. (2016). High-density linkage map construction and QTL analysis for earliness-related traits in *Gossypium hirsutum* L. BMC Genomics 17:909. 10.1186/s12864-016-3269-y 27835938 PMC5106845

[B11] Kosambi D. D. (1944). The estimation of map distances from recombination values. Ann. Eugen. 12, 172–175. 10.1111/j.1469-1809.1943.tb02321.x

[B12] Krzywinski M. Schein J. Birol I. Connors J. Gascoyne R. Horsman D.. (2009). Circos: an information aesthetic for comparative genomics. Genome Res. 19, 1639–1645. 10.1101/gr.092759.109 19541911 PMC2752132

[B13] Lander E. Kruglyak L. (1995). Genetic dissection of complex traits guidelines for interpreting and reporting linkage results. Nat. Genet. 11, 241–247. 10.1038/ng1195-241 7581446

[B14] Li C. Dong Y. Zhao T. Li L. Li C. Yu E.. (2016). Genome-wide SNP linkage mapping and QTL analysis for fiber quality and yield traits in the Upland Cotton recombinant inbred lines population. Front. Plant Sci. 7:1356. 10.3389/fpls.2016.01356 27660632 PMC5014859

[B15] Li F. Fan G. Lu C. Xiao G. Zou C. Kohel R. J.. (2015). Genome sequence of cultivated Upland cotton (*Gossypium hirsutum* TM-1) provides insights into genome evolution. Nat. Biotechnol. 33, 524–530. 10.1038/nbt.3208 25893780

[B16] Li F. Fan G. Wang K. Sun F. Yuan Y. Song G.. (2014). Genome sequence of the cultivated cotton *Gossypium arboreum*. Nat. Genet. 46, 567–572. 10.1038/ng.2987 24836287

[B17] Li X. Jin X. Wang H. Zhang X. Lin Z. (2016). Structure, evolution, and comparative genomics of tetraploid cotton based on a high-density genetic linkage map. DNA Res. 23, 283–293. 10.1093/dnares/dsw016 27084896 PMC4909315

[B18] Liu D. Liu F. Shan X. Zhang J. Tang S. Fang X.. (2015). Construction of a high-density genetic map and lint percentage and cottonseed nutrient trait QTL identification in upland cotton (*Gossypium hirsutum* L.). Mol. Genet. Genomics 290, 1683–1700. 10.1007/s00438-015-1027-5 25796191

[B19] Liu X. Teng Z. Wang J. Wu T. Zhang Z. Deng X.. (2017). Enriching an intraspecific genetic map and identifying QTL for fiber quality and yield component traits across multiple environments in Upland cotton (*Gossypium hirsutum* L.). Mol. Genet. Genomics. 292, 1281-1306. 10.1007/s00438-017-1347-8 28733817

[B20] Liu X. Zhao B. Zheng H. J. Hu Y. Lu G. Yang C. Q.. (2015). *Gossypium barbadense* genome sequence provides insight into the evolution of extra-long staple fiber and specialized metabolites. Sci. Rep. 5:14139. 10.1038/srep14139 26420475 PMC4588572

[B21] Montero-Pau J. Blanca J. Esteras C. Martínez-Pérez E. M. Gomez P. Monforte A. J.. (2017). An SNP-based saturated genetic map and QTL analysis of fruit-related traits in Zucchini using Genotyping-by-sequencing. BMC Genomics 18:94. 10.1186/s12864-016-3439-y 28100189 PMC5241963

[B22] Palanga K. K. Jamshed M. Rashid M. H. O. Gong J. Li J. Iqbal M. S.. (2017). Quantitative trait locus mapping for verticillium wilt resistance in an Upland Cotton recombinant inbred line using SNP-based high density genetic map. Front. Plant Sci. 8:382. 10.3389/fpls.2017.00382 28424708 PMC5380748

[B23] Paterson A. H. Lander E. S. Hewitt J. D. Peterson S. Lincoln S. E. Tanksley S. D. (1988). Resolution of quantitative traits into Mendelian factors by using a complete linkage map of restriction fragment length polymorphisms. Nature 335, 721–726. 10.1038/335721a0 2902517

[B24] Paterson A. H. Wendel J. F. Gundlach H. Guo H. Jenkins J. Jin D.. (2012). Repeated polyploidization of *Gossypium* genomes and the evolution of spinnable cotton fibres. Nature 492, 423–427. 10.1038/nature11798 23257886

[B25] Qi H. Wang N. Qiao W. Xu Q. Zhou H. Shi J. (2017). Construction of a high-density genetic map using genotyping by sequencing (GBS) for quantitative trait loci (QTL) analysis of three plant morphological traits in upland cotton (*Gossypium hirsutum* L.). Euphytica 213:83 10.1007/s10681-017-1867-7

[B26] Said J. I. Knapka J. A. Song M. Zhang J. (2015a). Cotton QTLdb: a cotton QTL database for QTL analysis, visualization, and comparison between *Gossypium hirsutum* and *G*. hirsutum x G. barbadense populations. Mol. Genet. Genomics 290, 1615–1625. 10.1007/s00438-015-1021-y 25758743

[B27] Said J. I. Lin Z. Zhang X. Song M. Zhang J. (2013). A comprehensive meta QTL analysis for fiber quality, yield, yield related and morphological traits, drought tolerance, and disease resistance in tetraploid cotton. BMC Genomics 14:776. 10.1186/1471-2164-14-776 24215677 PMC3830114

[B28] Said J. I. Song M. Wang H. Lin Z. Zhang X. Fang D. D.. (2015b). A comparative meta-analysis of QTL between intraspecific *Gossypium hirsutum* and interspecific *G*. hirsutum x G. barbadense populations. Mol. Genet. Genomics 290, 1003–1025. 10.1007/s00438-014-0963-9 25501533

[B29] Shao C. Niu Y. Rastas P. Liu Y. Xie Z. Li H.. (2015). Genome-wide SNP identification for the construction of a high-resolution genetic map of Japanese flounder (*Paralichthys olivaceus*): applications to QTL mapping of *Vibrio anguillarum* disease resistance and comparative genomic analysis. DNA Res. 22, 161–170. 10.1093/dnares/dsv001 25762582 PMC4401326

[B30] Shao Q. Zhang F. Tang S. Liu Y. Fang X. Liu D. (2014). Identifying QTL for fiber quality traits with three upland cotton (*Gossypium hirsutum* L.) populations. Euphytica 198, 43–58. 10.1007/s10681-014-1082-8

[B31] Shen X. Guo W. Lu Q. Zhu X. Yuan Y. Zhang T. (2007). Genetic mapping of quantitative trait loci for fiber quality and yield trait by RIL approach in Upland cotton. Euphytica 155, 371–380. 10.1007/s10681-006-9338-6

[B32] Shen X. Guo W. Zhu X. Yuan Y. Yu J. Z. Kohel R. J. (2005). Molecular mapping of QTLs for fiber qualities in three diverse lines in Upland cotton using SSR markers. Mol. Breed. 15, 169–181. 10.1007/s11032-004-4731-0

[B33] Sun F.-D. Zhang J.-H. Wang S.-F. Gong W.-K. Shi Y.-Z. Liu A.-Y. (2012). QTL mapping for fiber quality traits across multiple generations and environments in upland cotton. Mol. Breed. 30, 569–582. 10.1007/s11032-011-9645-z

[B34] Sun Z. Wang X. Liu Z. Gu Q. Zhang Y. Li Z.. (2017). Genome-wide association study discovered genetic variation and candidate genes of fibre quality traits in *Gossypium hirsutum* L. Plant Biotechnol. J. 15, 982–996. 10.1111/pbi.12693 28064470 PMC5506648

[B35] Tan Z. Fang X. Tang S. Zhang J. Liu D. Teng Z. (2014). Genetic map and QTL controlling fiber quality traits in upland cotton (*Gossypium hirsutum* L.). Euphytica 203, 615–628. 10.1007/s10681-014-1288-9

[B36] Tang S. Teng Z. Zhai T. Fang X. Liu F. Liu D. (2014). Construction of genetic map and QTL analysis of fiber quality traits for Upland cotton (*Gossypium hirsutum* L.). Euphytica 201, 195–213. 10.1007/s10681-014-1189-y

[B37] Taylor D. R. Ingvarsson P. K. (2003). Common features of segregation distortion in plants and animals. Genetica 117, 27–35. 10.1023/A:1022308414864 12656570

[B38] Truco M. J. Ashrafi H. Kozik A. Leeuwen H. V. Bowers J. Wo S. R. C.. (2013). An Ultra-high-density, transcript-based, genetic map of lettuce. G3 3, 617–631. 10.1534/g3.112.004929 23550116 PMC3618349

[B39] Van Ooijen J. W. (2006). JoinMap 4.0, Software for the Calculation of Genetic Linkage Maps in Experimental Populations. Wageningen: Kyazma, B.V.

[B40] Van Ooijen J. W. (2009). MapQTL 6.0. Software for the Mapping of Quantitative Trait Loci in Experimental Populations. Wageningen: Kyazma, B.V.

[B41] Voorrips R. E. (2002). MapChart: software for the graphical presentation of linkage maps and QTLs. J. Hered. 93, 77–78. 10.1093/jhered/93.1.77 12011185

[B42] Wang C. Chen S. Yu S. (2011). Functional markers developed from multiple loci in GS3 for fine marker-assisted selection of grain length in rice. Theor. Appl. Genet. 122, 905–913. 10.1007/s00122-010-1497-0 21107518

[B43] Wang H. Jin X. Zhang B. Shen C. Lin Z. (2015). Enrichment of an intraspecific genetic map of upland cotton by developing markers using parental RAD sequencing. DNA Res. 22, 147–160. 10.1093/dnares/dsu047 25656006 PMC4401325

[B44] Wang K. Wang Z. Li F. Ye W. Wang J. Song G.. (2012). The draft genome of a diploid cotton *Gossypium raimondii*. Nat. Genet. 44, 1098–1103. 10.1038/ng.2371 22922876

[B45] Wang Y. Ning Z. Hu Y. Chen J. Zhao R. Chen H.. (2015). Molecular mapping of restriction-site associated DNA markers in allotetraploid Upland Cotton. PLoS ONE 10:e0124781. 10.1371/journal.pone.0124781 25894395 PMC4403916

[B46] Xian-Liang S. Xue-Zhen S. Tian-Zhen Z. (2007). Segregation distortion and its effect on genetic mapping in plants. J. Agric. Biotechnol. 3, 163–169. 10.1079/CJB2006110

[B47] Xu Y. Crouch J. H. (2008). Marker-assisted selection in plant breeding: from publications to practice. Crop Sci. 48, 391-407. 10.2135/cropsci2007.04.0191

[B48] Yang H. Li C. Lam H. M. Clements J. Yan G. Zhao S. (2015). Sequencing consolidates molecular markers with plant breeding practice. Theor. Appl. Genet. 128, 779–795. 10.1007/s00122-015-2499-8 25821196

[B49] Yu Y. Yuan D. Liang S. Li X. Wang X. Lin Z. (2011). Genome structure of cotton revealed by a genome- wide SSR genetic map constructed from a BC1 population between *Gossypium hirsutum* and *G. barbadense*. BMC Genomics. 12:15. 10.1186/1471-2164-12-15 21214949 PMC3031231

[B50] Yuan D. Tang Z. Wang M. Gao W. Tu L. Jin X.. (2015). The genome sequence of Sea-Island cotton (*Gossypium barbadense*) provides insights into the allopolyploidization and development of superior spinnable fibres. Sci. Rep. 5:17662. 10.1038/srep17662 26634818 PMC4669482

[B51] Zhang P. He Z. Tian X. Gao F. Xu D. Liu J. (2017). Cloning of TaTPP-6AL1 associated with grain weight in bread wheat and development of functional marker. Mol. Breed. 37:78 10.1007/s11032-017-0676-y

[B52] Zhang T. Hu Y. Jiang W. Fang L. Guan X. Chen J.. (2015). Sequencing of allotetraploid cotton (*Gossypium hirsutum* L. acc. TM-1) provides a resource for fiber improvement. Nat. Biotechnol. 33, 531-537. 10.1038/nbt.3207 25893781

[B53] Zhang Z. Ge Q. Liu A. Li J. Gong J. Shang H. (2017). Construction of a high-density genetic map and its application to QTL identification for fiber strength in Upland Cotton. Crop Sci. 57:774 10.2135/cropsci2016.06.0544

[B54] Zhang Z. Shang H. Shi Y. Huang L. Li J. Ge Q.. (2016). Construction of a high-density genetic map by specific locus amplified fragment sequencing (SLAF-seq) and its application to Quantitative Trait Loci (QTL) analysis for boll weight in upland cotton (*Gossypium hirsutum*.). BMC Plant Biol. 16:79. 10.1186/s12870-016-0741-4 27067834 PMC4827241

[B55] Zhang Z.-S. Hu M.-C. Zhang J. Liu D.-J. Zheng J. Zhang K. (2009). Construction of a comprehensive PCR-based marker linkage map and QTL mapping for fiber quality traits in upland cotton (*Gossypium hirsutum* L.). Mol. Breed. 24, 49–61. 10.1007/s11032-009-9271-1

[B56] Zhang Z.-S. Xiao Y.-H. Luo M. Li X.-B. Luo X.-Y. Hou L. (2005). Construction of a genetic linkage map and QTL analysis of fiber-related traits in upland cotton (*Gossypium hirsutum* L.). Euphytica 144, 91–99. 10.1007/s10681-005-4629-x

