# Supplementary material for: Genetic Map Construction and Fiber Quality QTL Mapping Using the CottonSNP80K Array in Upland Cotton
Source: Front Plant Sci. 2018 Feb 27;9:225. doi: 10.3389/fpls.2018.00225 (PMC5835031; doi:10.3389/fpls.2018.00225)
Supplement: Supplementary file 5 [file Image1.pdf]

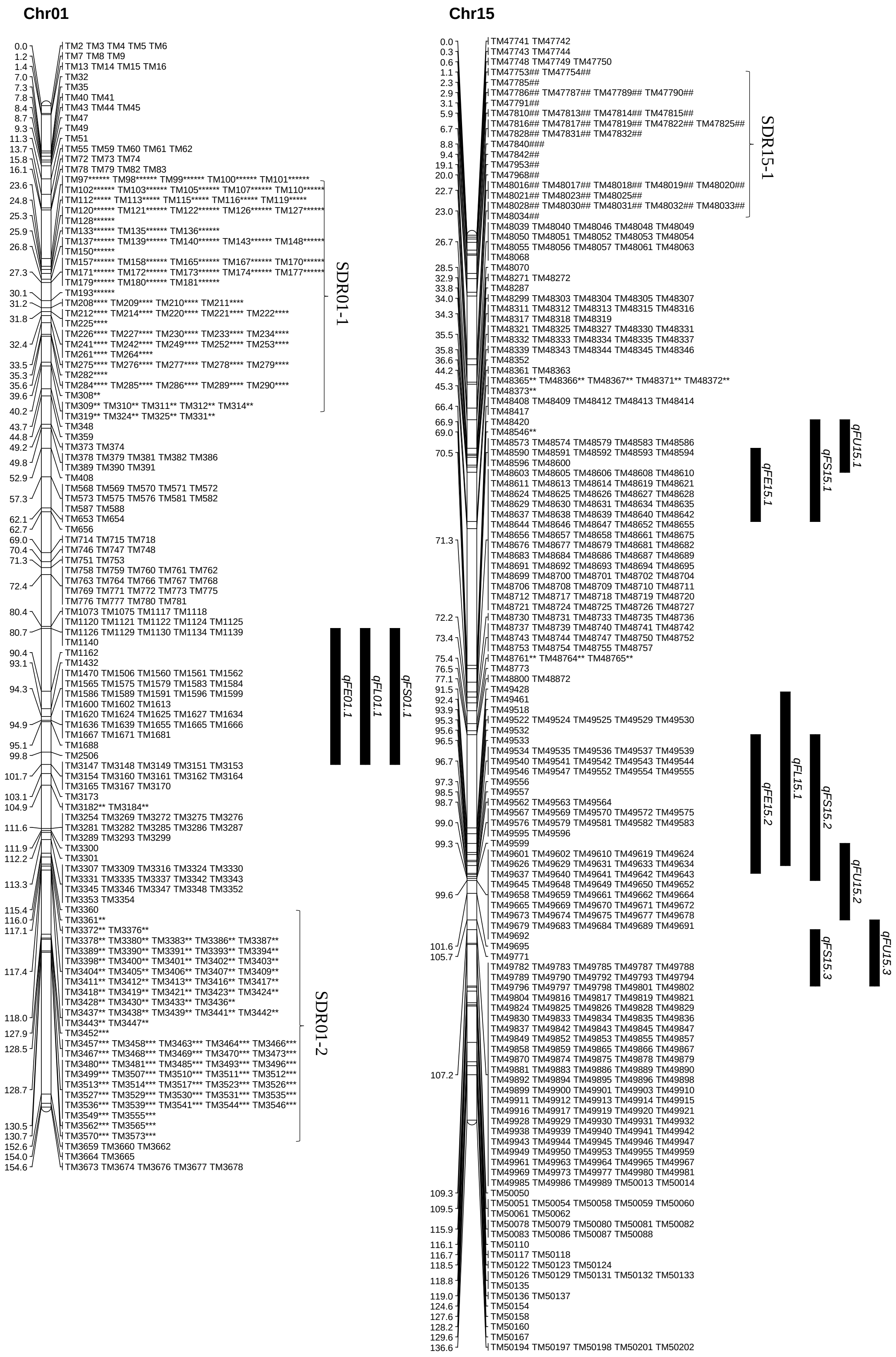
Chr01
0.0
1.2
1.4
7.0
7.3
7.8
8.4
8.7
9.3
11.3
13.7
15.8
16.1
23.6
24.8
25.3
25.9
26.8
27.3
30.1
31.2
31.8
32.4
33.5
35.3
35.6
39.6
40.2
43.7
44.8
49.2
49.8
52.9
57.3
62.1
62.7
69.0
70.4
71.3
72.4
80.4
80.7
90.4
93.1
94.3
94.9
95.1
99.8
101.7
103.1
104.9
111.6
111.9
112.2
113.3
115.4
116.0
117.1
117.4
118.0
127.9
128.5
128.7
130.5
130.7
152.6
154.0
154.6
TM2 TM3 TM4 TM5 TM6
TM7 TM8 TM9
TM13 TM14 TM15 TM16
TM32
TM35
TM40 TM41
TM43 TM44 TM45
TM47
TM49
TM51
TM55 TM59 TM60 TM61 TM62
TM72 TM73 TM74
TM78 TM79 TM82 TM83
TM97****** TM98****** TM99****** TM100****** TM101******
TM102****** TM103****** TM105****** TM107****** TM110******
TM112***** TM113***** TM115***** TM116***** TM119*****
TM120****** TM121****** TM122****** TM126****** TM127******
TM128******
TM133****** TM135****** TM136******
TM137****** TM139****** TM140****** TM143****** TM148******
TM150******
TM157****** TM158****** TM165****** TM167****** TM170******
TM171****** TM172****** TM173****** TM174****** TM177******
TM179****** TM180****** TM181******
TM193******
TM208**** TM209**** TM210**** TM211****
TM212**** TM214**** TM220**** TM221**** TM222****
TM225****
TM226**** TM227**** TM230**** TM233**** TM234****
TM241**** TM242**** TM249**** TM252**** TM253****
TM261**** TM264****
TM275**** TM276**** TM277**** TM278**** TM279****
TM282****
TM284**** TM285**** TM286**** TM289**** TM290****
TM308**
TM309** TM310** TM311** TM312** TM314**
TM319** TM324** TM325** TM331**
TM348
TM359
TM373 TM374
TM378 TM379 TM381 TM382 TM386
TM389 TM390 TM391
TM408
TM568 TM569 TM570 TM571 TM572
TM573 TM575 TM576 TM581 TM582
TM587 TM588
TM653 TM654
TM656
TM714 TM715 TM718
TM746 TM747 TM748
TM751 TM753
TM758 TM759 TM760 TM761 TM762
TM763 TM764 TM766 TM767 TM768
TM769 TM771 TM772 TM773 TM775
TM776 TM777 TM780 TM781
TM1073 TM1075 TM1117 TM1118
TM1120 TM1121 TM1122 TM1124 TM1125
TM1126 TM1129 TM1130 TM1134 TM1139
TM1140
TM1162
TM1432
TM1470 TM1506 TM1560 TM1561 TM1562
TM1565 TM1575 TM1579 TM1583 TM1584
TM1586 TM1589 TM1591 TM1596 TM1599
TM1600 TM1602 TM1613
TM1620 TM1624 TM1625 TM1627 TM1634
TM1636 TM1639 TM1655 TM1665 TM1666
TM1667 TM1671 TM1681
TM1688
TM2506
TM3147 TM3148 TM3149 TM3151 TM3153
TM3154 TM3160 TM3161 TM3162 TM3164
TM3165 TM3167 TM3170
TM3173
TM3182** TM3184**
TM3254 TM3269 TM3272 TM3275 TM3276
TM3281 TM3282 TM3285 TM3286 TM3287
TM3289 TM3293 TM3299
TM3300
TM3301
TM3307 TM3309 TM3316 TM3324 TM3330
TM3331 TM3335 TM3337 TM3342 TM3343
TM3345 TM3346 TM3347 TM3348 TM3352
TM3353 TM3354
TM3360
TM3361**
TM3372** TM3376**
TM3378** TM3380** TM3383** TM3386** TM3387**
TM3389** TM3390** TM3391** TM3393** TM3394**
TM3398** TM3400** TM3401** TM3402** TM3403**
TM3404** TM3405** TM3406** TM3407** TM3409**
TM3411** TM3412** TM3413** TM3416** TM3417**
TM3418** TM3419** TM3421** TM3423** TM3424**
TM3428** TM3430** TM3433** TM3436**
TM3437** TM3438** TM3439** TM3441** TM3442**
TM3443** TM3447**
TM3452***
TM3457*** TM3458*** TM3463*** TM3464*** TM3466***
TM3467*** TM3468*** TM3469*** TM3470*** TM3473***
TM3480*** TM3481*** TM3485*** TM3493*** TM3496***
TM3499*** TM3507*** TM3510*** TM3511*** TM3512***
TM3513*** TM3514*** TM3517*** TM3523*** TM3526***
TM3527*** TM3529*** TM3530*** TM3531*** TM3535***
TM3536*** TM3539*** TM3541*** TM3544*** TM3546***
TM3549*** TM3555***
TM3562*** TM3565***
TM3570*** TM3573***
TM3659 TM3660 TM3662
TM3664 TM3665
TM3673 TM3674 TM3676 TM3677 TM3678
SDR01-1
SDR01-2
qFE01.1
qFL01.1
qFS01.1
Chr15
0.0
0.3
0.6
1.1
2.3
2.9
3.1
5.9
6.7
8.8
9.4
19.1
20.0
22.7
23.0
26.7
28.5
32.9
33.8
34.0
34.3
35.5
35.8
36.6
44.2
45.3
66.4
66.9
69.0
70.5
71.3
72.2
73.4
75.4
76.5
77.1
91.5
92.4
93.9
95.3
95.6
96.5
96.7
97.3
98.5
98.7
99.0
99.3
99.6
101.6
105.7
107.2
109.3
109.5
115.9
116.1
116.7
118.5
118.8
119.0
124.6
127.6
128.2
129.6
136.6
TM47741 TM47742
TM47743 TM47744
TM47748 TM47749 TM47750
TM47753## TM47754##
TM47785##
TM47786## TM47787## TM47789## TM47790##
TM47791##
TM47810## TM47813## TM47814## TM47815##
TM47816## TM47817## TM47819## TM47822## TM47825##
TM47828## TM47831## TM47832##
TM47840###
TM47842##
TM47953##
TM47968##
TM48016## TM48017## TM48018## TM48019## TM48020##
TM48021## TM48023## TM48025##
TM48028## TM48030## TM48031## TM48032## TM48033##
TM48034##
TM48039 TM48040 TM48046 TM48048 TM48049
TM48050 TM48051 TM48052 TM48053 TM48054
TM48055 TM48056 TM48057 TM48061 TM48063
TM48068
TM48070
TM48271 TM48272
TM48287
TM48299 TM48303 TM48304 TM48305 TM48307
TM48311 TM48312 TM48313 TM48315 TM48316
TM48317 TM48318 TM48319
TM48321 TM48325 TM48327 TM48330 TM48331
TM48332 TM48333 TM48334 TM48335 TM48337
TM48339 TM48343 TM48344 TM48345 TM48346
TM48352
TM48361 TM48363
TM48365** TM48366** TM48367** TM48371** TM48372**
TM48373**
TM48408 TM48409 TM48412 TM48413 TM48414
TM48417
TM48420
TM48546**
TM48573 TM48574 TM48579 TM48583 TM48586
TM48590 TM48591 TM48592 TM48593 TM48594
TM48596 TM48600
TM48603 TM48605 TM48606 TM48608 TM48610
TM48611 TM48613 TM48614 TM48619 TM48621
TM48624 TM48625 TM48626 TM48627 TM48628
TM48629 TM48630 TM48631 TM48634 TM48635
TM48637 TM48638 TM48639 TM48640 TM48642
TM48644 TM48646 TM48647 TM48652 TM48655
TM48656 TM48657 TM48658 TM48661 TM48675
TM48676 TM48677 TM48679 TM48681 TM48682
TM48683 TM48684 TM48686 TM48687 TM48689
TM48691 TM48692 TM48693 TM48694 TM48695
TM48699 TM48700 TM48701 TM48702 TM48704
TM48706 TM48708 TM48709 TM48710 TM48711
TM48712 TM48717 TM48718 TM48719 TM48720
TM48721 TM48724 TM48725 TM48726 TM48727
TM48730 TM48731 TM48733 TM48735 TM48736
TM48737 TM48739 TM48740 TM48741 TM48742
TM48743 TM48744 TM48747 TM48750 TM48752
TM48753 TM48754 TM48755 TM48757
TM48761** TM48764** TM48765**
TM48773
TM48800 TM48872
TM49428
TM49461
TM49518
TM49522 TM49524 TM49525 TM49529 TM49530
TM49532
TM49533
TM49534 TM49535 TM49536 TM49537 TM49539
TM49540 TM49541 TM49542 TM49543 TM49544
TM49546 TM49547 TM49552 TM49554 TM49555
TM49556
TM49557
TM49562 TM49563 TM49564
TM49567 TM49569 TM49570 TM49572 TM49575
TM49576 TM49579 TM49581 TM49582 TM49583
TM49595 TM49596
TM49599
TM49601 TM49602 TM49610 TM49619 TM49624
TM49626 TM49629 TM49631 TM49633 TM49634
TM49637 TM49640 TM49641 TM49642 TM49643
TM49645 TM49648 TM49649 TM49650 TM49652
TM49658 TM49659 TM49661 TM49662 TM49664
TM49665 TM49669 TM49670 TM49671 TM49672
TM49673 TM49674 TM49675 TM49677 TM49678
TM49679 TM49683 TM49684 TM49689 TM49691
TM49692
TM49695
TM49771
TM49782 TM49783 TM49785 TM49787 TM49788
TM49789 TM49790 TM49792 TM49793 TM49794
TM49796 TM49797 TM49798 TM49801 TM49802
TM49804 TM49816 TM49817 TM49819 TM49821
TM49824 TM49825 TM49826 TM49828 TM49829
TM49830 TM49833 TM49834 TM49835 TM49836
TM49837 TM49842 TM49843 TM49845 TM49847
TM49849 TM49853 TM49854 TM49855 TM49857
TM49858 TM49859 TM49865 TM49866 TM49867
TM49870 TM49874 TM49875 TM49878 TM49879
TM49881 TM49883 TM49886 TM49889 TM49890
TM49892 TM49894 TM49895 TM49896 TM49898
TM49899 TM49900 TM49901 TM49903 TM49910
TM49911 TM49912 TM49913 TM49914 TM49915
TM49916 TM49917 TM49919 TM49920 TM49921
TM49928 TM49929 TM49930 TM49931 TM49932
TM49938 TM49939 TM49940 TM49941 TM49942
TM49943 TM49944 TM49945 TM49946 TM49947
TM49949 TM49950 TM49953 TM49955 TM49959
TM49961 TM49963 TM49964 TM49965 TM49967
TM49969 TM49973 TM49977 TM49980 TM49981
TM49985 TM49986 TM49989 TM50013 TM50014
TM50050
TM50051 TM50054 TM50058 TM50059 TM50060
TM50061 TM50062
TM50078 TM50079 TM50080 TM50081 TM50082
TM50083 TM50086 TM50087 TM50088
TM50110
TM50117 TM50118
TM50122 TM50123 TM50124
TM50126 TM50129 TM50131 TM50132 TM50133
TM50135
TM50136 TM50137
TM50154
TM50158
TM50160
TM50167
TM50194 TM50197 TM50198 TM50201 TM50202
SDR15-1
qFU15.1
qFS15.1
qFE15.1
qFL15.1
qFE15.2
qFS15.2
qFU15.2
qFS15.3
qFU15.3

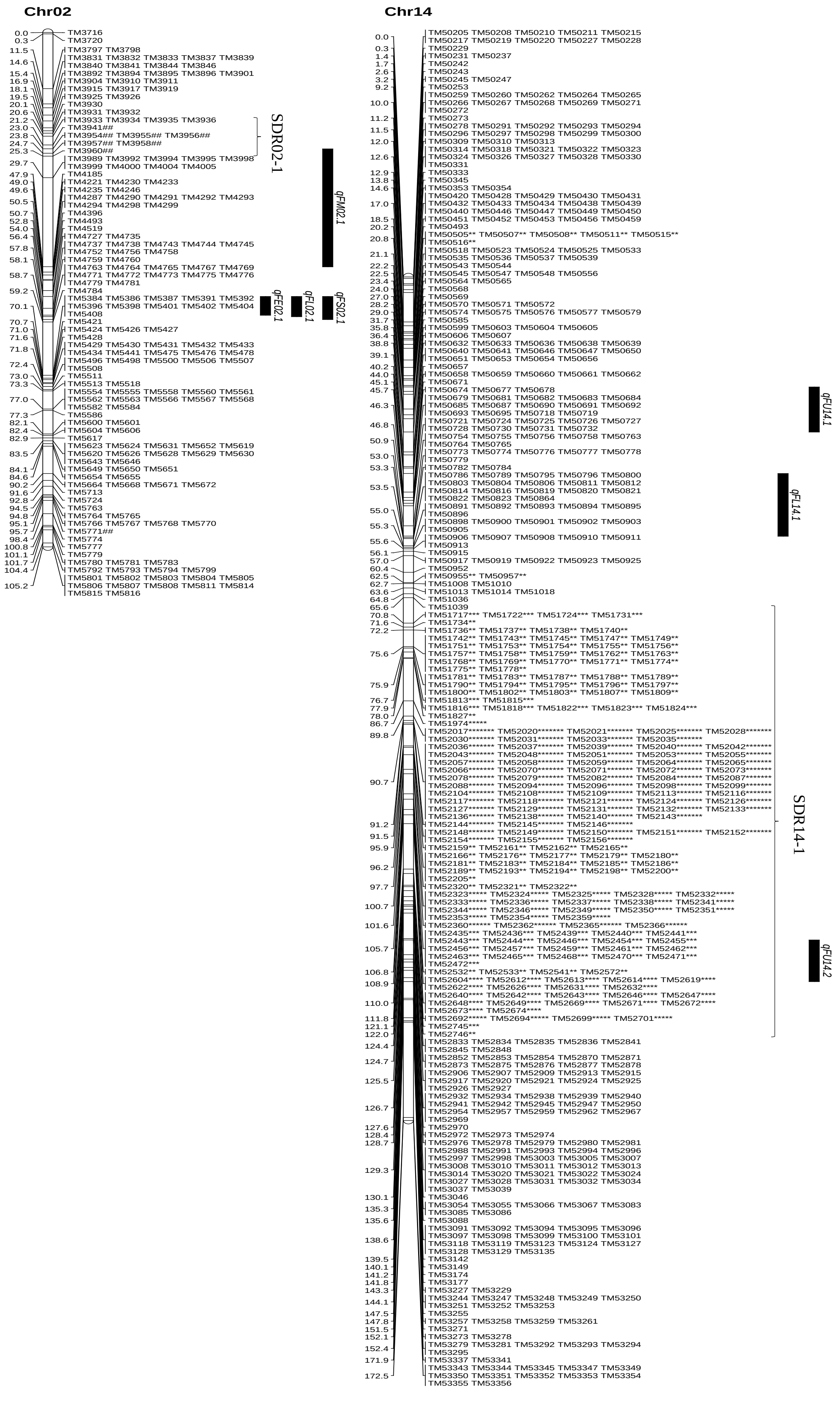
Chr02
Chr14
SDR02-1
qFM02.1
qFE02.1
qFL02.1
qFS02.1
SDR14-1
qFU14.1
qFL14.1
qFU14.2

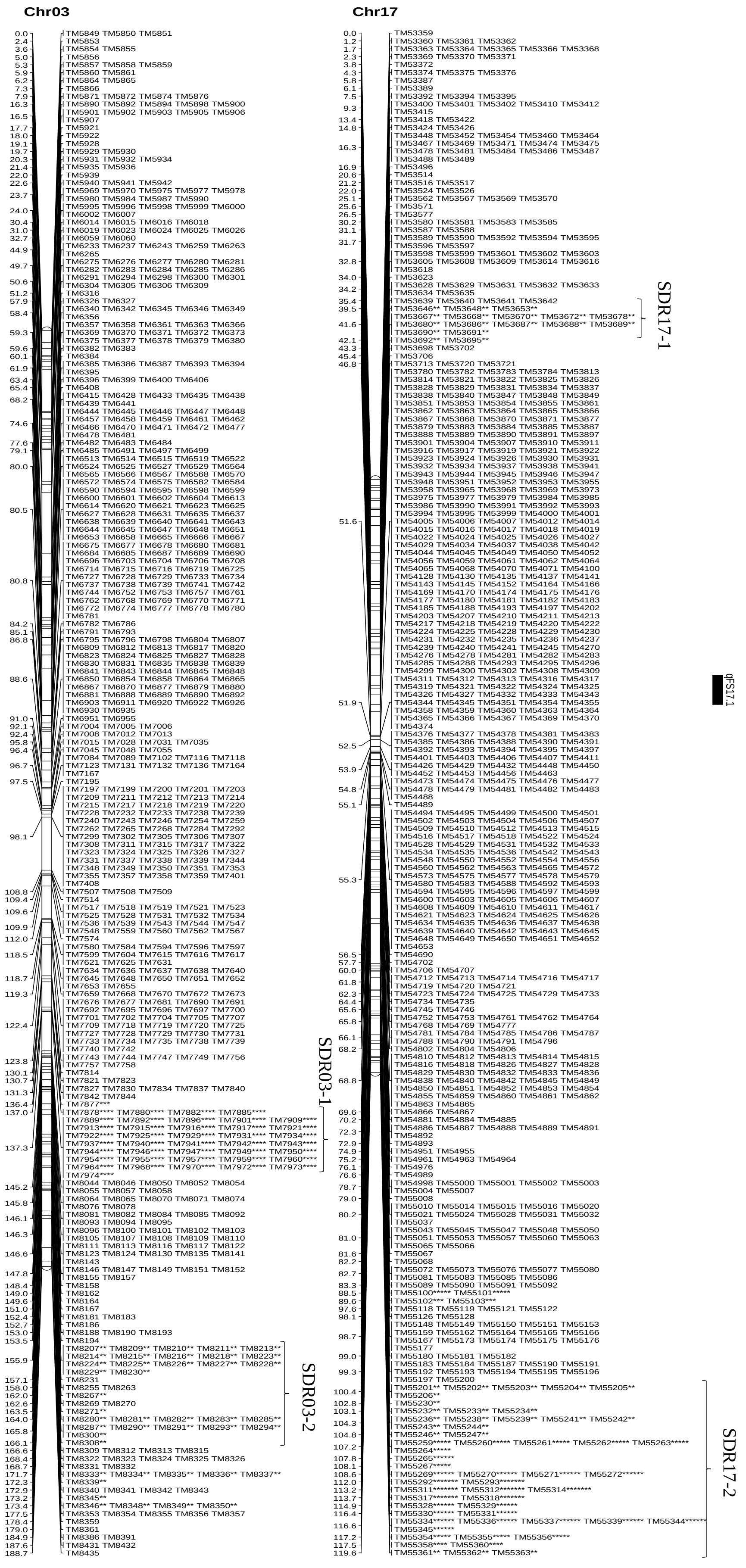
Chr03
Chr17
SDR17-1
qFS17.1
SDR03-1
SDR03-2
SDR17-2

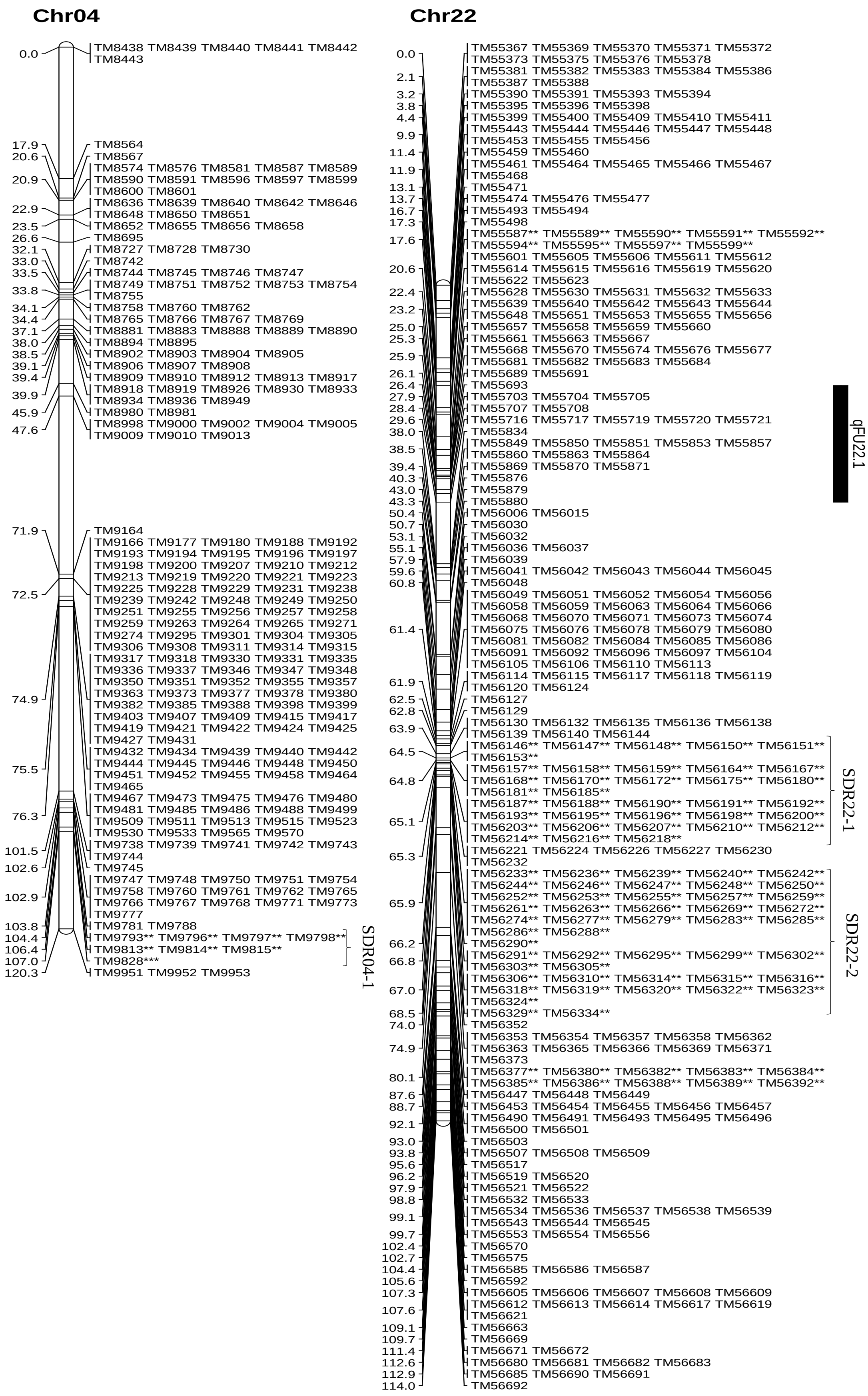
Chr04
0.0
17.9
20.6
20.9
22.9
23.5
26.6
32.1
33.0
33.5
33.8
34.1
34.4
37.1
38.0
38.5
39.1
39.4
39.9
45.9
47.6
71.9
72.5
74.9
75.5
76.3
101.5
102.6
102.9
103.8
104.4
106.4
107.0
120.3
TM8438 TM8439 TM8440 TM8441 TM8442
TM8443
TM8564
TM8567
TM8574 TM8576 TM8581 TM8587 TM8589
TM8590 TM8591 TM8596 TM8597 TM8599
TM8600 TM8601
TM8636 TM8639 TM8640 TM8642 TM8646
TM8648 TM8650 TM8651
TM8652 TM8655 TM8656 TM8658
TM8695
TM8727 TM8728 TM8730
TM8742
TM8744 TM8745 TM8746 TM8747
TM8749 TM8751 TM8752 TM8753 TM8754
TM8755
TM8758 TM8760 TM8762
TM8765 TM8766 TM8767 TM8769
TM8881 TM8883 TM8888 TM8889 TM8890
TM8894 TM8895
TM8902 TM8903 TM8904 TM8905
TM8906 TM8907 TM8908
TM8909 TM8910 TM8912 TM8913 TM8917
TM8918 TM8919 TM8926 TM8930 TM8933
TM8934 TM8936 TM8949
TM8980 TM8981
TM8998 TM9000 TM9002 TM9004 TM9005
TM9009 TM9010 TM9013
TM9164
TM9166 TM9177 TM9180 TM9188 TM9192
TM9193 TM9194 TM9195 TM9196 TM9197
TM9198 TM9200 TM9207 TM9210 TM9212
TM9213 TM9219 TM9220 TM9221 TM9223
TM9225 TM9228 TM9229 TM9231 TM9238
TM9239 TM9242 TM9248 TM9249 TM9250
TM9251 TM9255 TM9256 TM9257 TM9258
TM9259 TM9263 TM9264 TM9265 TM9271
TM9274 TM9295 TM9301 TM9304 TM9305
TM9306 TM9308 TM9311 TM9314 TM9315
TM9317 TM9318 TM9330 TM9331 TM9335
TM9336 TM9337 TM9346 TM9347 TM9348
TM9350 TM9351 TM9352 TM9355 TM9357
TM9363 TM9373 TM9377 TM9378 TM9380
TM9382 TM9385 TM9388 TM9398 TM9399
TM9403 TM9407 TM9409 TM9415 TM9417
TM9419 TM9421 TM9422 TM9424 TM9425
TM9427 TM9431
TM9432 TM9434 TM9439 TM9440 TM9442
TM9444 TM9445 TM9446 TM9448 TM9450
TM9451 TM9452 TM9455 TM9458 TM9464
TM9465
TM9467 TM9473 TM9475 TM9476 TM9480
TM9481 TM9485 TM9486 TM9488 TM9499
TM9509 TM9511 TM9513 TM9515 TM9523
TM9530 TM9533 TM9565 TM9570
TM9738 TM9739 TM9741 TM9742 TM9743
TM9744
TM9745
TM9747 TM9748 TM9750 TM9751 TM9754
TM9758 TM9760 TM9761 TM9762 TM9765
TM9766 TM9767 TM9768 TM9771 TM9773
TM9777
TM9781 TM9788
TM9793** TM9796** TM9797** TM9798**
TM9813** TM9814** TM9815**
TM9828***
TM9951 TM9952 TM9953
SDR04-1
Chr22
0.0
2.1
3.2
3.8
4.4
9.9
11.4
11.9
13.1
13.7
16.7
17.3
17.6
20.6
22.4
23.2
25.0
25.3
25.9
26.1
26.4
27.9
28.4
29.6
38.0
38.5
39.4
40.3
43.0
43.3
50.4
50.7
53.1
55.1
57.9
59.6
60.8
61.4
61.9
62.5
62.8
63.9
64.5
64.8
65.1
65.3
65.9
66.2
66.8
67.0
68.5
74.0
74.9
80.1
87.6
88.7
92.1
93.0
93.8
95.6
96.2
97.9
98.8
99.1
99.7
102.4
102.7
104.4
105.6
107.3
107.6
109.1
109.7
111.4
112.6
112.9
114.0
TM55367 TM55369 TM55370 TM55371 TM55372
TM55373 TM55375 TM55376 TM55378
TM55381 TM55382 TM55383 TM55384 TM55386
TM55387 TM55388
TM55390 TM55391 TM55393 TM55394
TM55395 TM55396 TM55398
TM55399 TM55400 TM55409 TM55410 TM55411
TM55443 TM55444 TM55446 TM55447 TM55448
TM55453 TM55455 TM55456
TM55459 TM55460
TM55461 TM55464 TM55465 TM55466 TM55467
TM55468
TM55471
TM55474 TM55476 TM55477
TM55493 TM55494
TM55498
TM55587** TM55589** TM55590** TM55591** TM55592**
TM55594** TM55595** TM55597** TM55599**
TM55601 TM55605 TM55606 TM55611 TM55612
TM55614 TM55615 TM55616 TM55619 TM55620
TM55622 TM55623
TM55628 TM55630 TM55631 TM55632 TM55633
TM55639 TM55640 TM55642 TM55643 TM55644
TM55648 TM55651 TM55653 TM55655 TM55656
TM55657 TM55658 TM55659 TM55660
TM55661 TM55663 TM55667
TM55668 TM55670 TM55674 TM55676 TM55677
TM55681 TM55682 TM55683 TM55684
TM55689 TM55691
TM55693
TM55703 TM55704 TM55705
TM55707 TM55708
TM55716 TM55717 TM55719 TM55720 TM55721
TM55834
TM55849 TM55850 TM55851 TM55853 TM55857
TM55860 TM55863 TM55864
TM55869 TM55870 TM55871
TM55876
TM55879
TM55880
TM56006 TM56015
TM56030
TM56032
TM56036 TM56037
TM56039
TM56041 TM56042 TM56043 TM56044 TM56045
TM56048
TM56049 TM56051 TM56052 TM56054 TM56056
TM56058 TM56059 TM56063 TM56064 TM56066
TM56068 TM56070 TM56071 TM56073 TM56074
TM56075 TM56076 TM56078 TM56079 TM56080
TM56081 TM56082 TM56084 TM56085 TM56086
TM56091 TM56092 TM56096 TM56097 TM56104
TM56105 TM56106 TM56110 TM56113
TM56114 TM56115 TM56117 TM56118 TM56119
TM56120 TM56124
TM56127
TM56129
TM56130 TM56132 TM56135 TM56136 TM56138
TM56139 TM56140 TM56144
TM56146** TM56147** TM56148** TM56150** TM56151**
TM56153**
TM56157** TM56158** TM56159** TM56164** TM56167**
TM56168** TM56170** TM56172** TM56175** TM56180**
TM56181** TM56185**
TM56187** TM56188** TM56190** TM56191** TM56192**
TM56193** TM56195** TM56196** TM56198** TM56200**
TM56203** TM56206** TM56207** TM56210** TM56212**
TM56214** TM56216** TM56218**
TM56221 TM56224 TM56226 TM56227 TM56230
TM56232
TM56233** TM56236** TM56239** TM56240** TM56242**
TM56244** TM56246** TM56247** TM56248** TM56250**
TM56252** TM56253** TM56255** TM56257** TM56259**
TM56261** TM56263** TM56266** TM56269** TM56272**
TM56274** TM56277** TM56279** TM56283** TM56285**
TM56286** TM56288**
TM56290**
TM56291** TM56292** TM56295** TM56299** TM56302**
TM56303** TM56305**
TM56306** TM56310** TM56314** TM56315** TM56316**
TM56318** TM56319** TM56320** TM56322** TM56323**
TM56324**
TM56329** TM56334**
TM56352
TM56353 TM56354 TM56357 TM56358 TM56362
TM56363 TM56365 TM56366 TM56369 TM56371
TM56373
TM56377** TM56380** TM56382** TM56383** TM56384**
TM56385** TM56386** TM56388** TM56389** TM56392**
TM56447 TM56448 TM56449
TM56453 TM56454 TM56455 TM56456 TM56457
TM56490 TM56491 TM56493 TM56495 TM56496
TM56500 TM56501
TM56503
TM56507 TM56508 TM56509
TM56517
TM56519 TM56520
TM56521 TM56522
TM56532 TM56533
TM56534 TM56536 TM56537 TM56538 TM56539
TM56543 TM56544 TM56545
TM56553 TM56554 TM56556
TM56570
TM56575
TM56585 TM56586 TM56587
TM56592
TM56605 TM56606 TM56607 TM56608 TM56609
TM56612 TM56613 TM56614 TM56617 TM56619
TM56621
TM56663
TM56669
TM56671 TM56672
TM56680 TM56681 TM56682 TM56683
TM56685 TM56690 TM56691
TM56692
qFU22.1
SDR22-1
SDR22-2

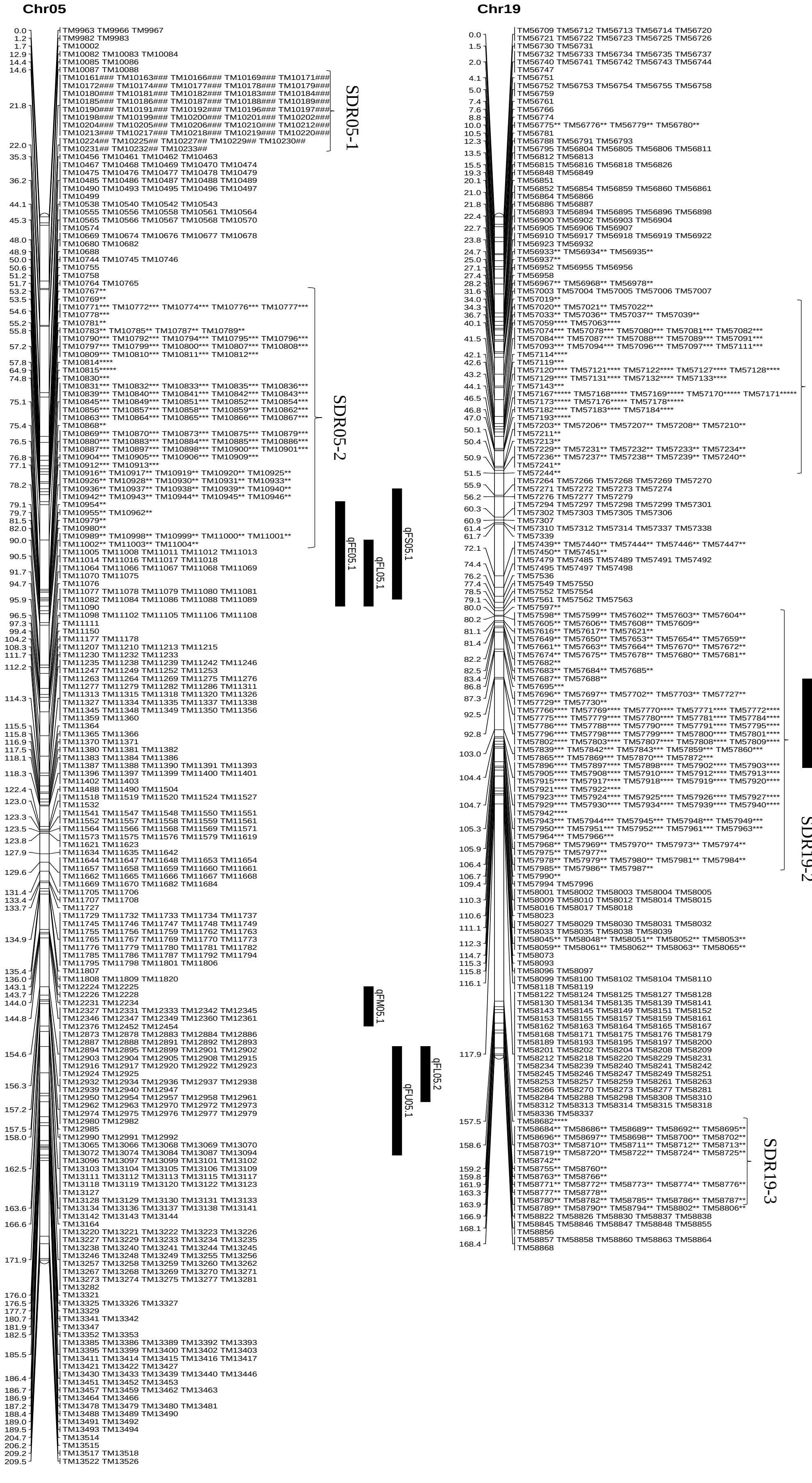
Chr05
Chr19
SDR05-1
SDR05-2
qFE05.1
qFL05.1
qFS05.1
qFM05.1
qFL05.2
qFU05.1
SDR19-1
qFM19.1
qFS19.1
qFL19.1
SDR19-2
qFS19.2
SDR19-3

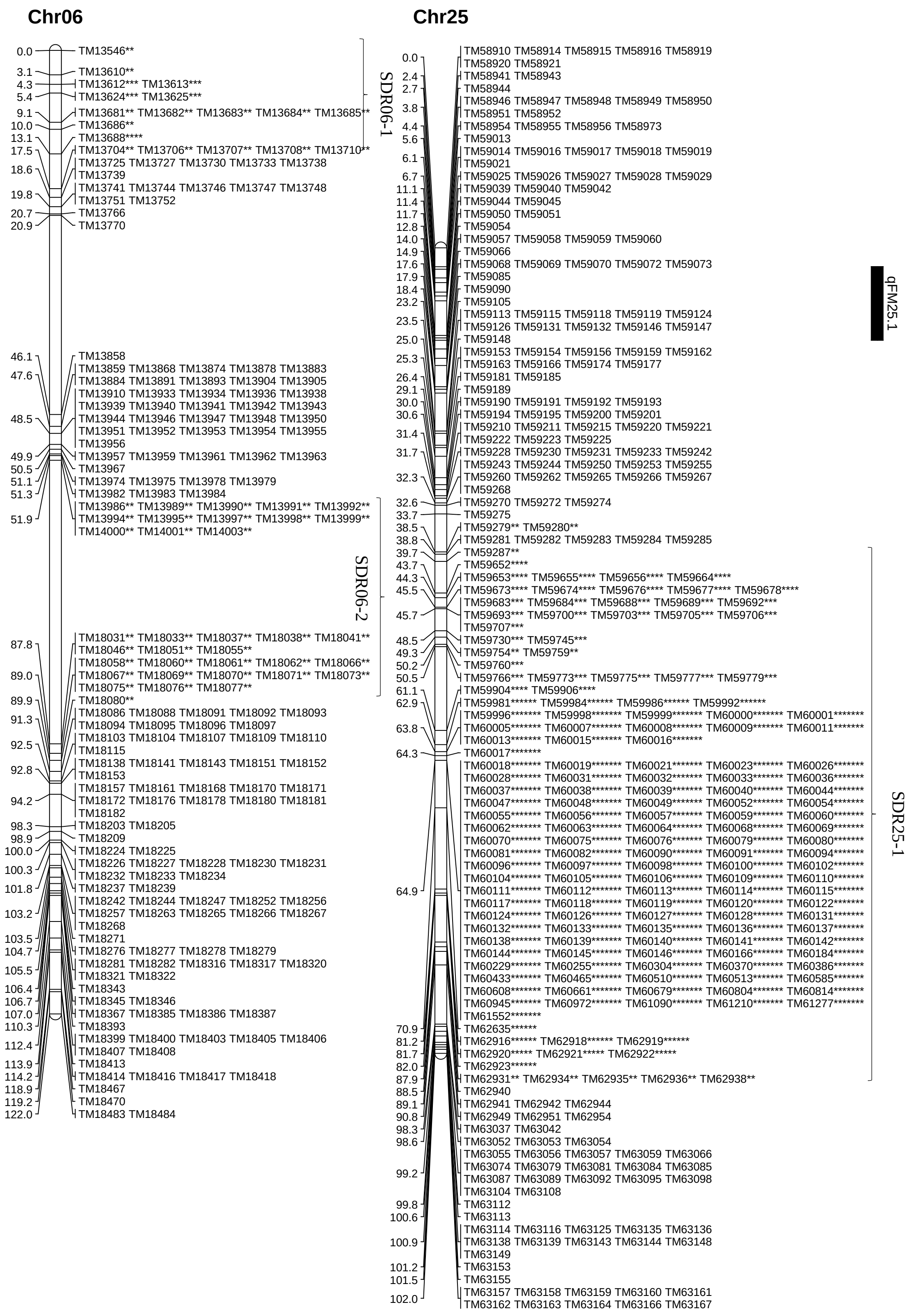

Chr06
Chr25
SDR06-1
SDR06-2
SDR25-1
qFM25.1
0.0 TM13546**
3.1 TM13610**
4.3 TM13612*** TM13613***
5.4 TM13624*** TM13625***
9.1 TM13681** TM13682** TM13683** TM13684** TM13685**
10.0 TM13686**
13.1 TM13688****
17.5 TM13704** TM13706** TM13707** TM13708** TM13710**
18.6 TM13725 TM13727 TM13730 TM13733 TM13738
TM13739
19.8 TM13741 TM13744 TM13746 TM13747 TM13748
TM13751 TM13752
20.7 TM13766
20.9 TM13770
46.1 TM13858
47.6 TM13859 TM13868 TM13874 TM13878 TM13883
TM13884 TM13891 TM13893 TM13904 TM13905
TM13910 TM13933 TM13934 TM13936 TM13938
TM13939 TM13940 TM13941 TM13942 TM13943
48.5 TM13944 TM13946 TM13947 TM13948 TM13950
TM13951 TM13952 TM13953 TM13954 TM13955
TM13956
49.9 TM13957 TM13959 TM13961 TM13962 TM13963
50.5 TM13967
51.1 TM13974 TM13975 TM13978 TM13979
51.3 TM13982 TM13983 TM13984
51.9 TM13986** TM13989** TM13990** TM13991** TM13992**
TM13994** TM13995** TM13997** TM13998** TM13999**
TM14000** TM14001** TM14003**
87.8 TM18031** TM18033** TM18037** TM18038** TM18041**
TM18046** TM18051** TM18055**
89.0 TM18058** TM18060** TM18061** TM18062** TM18066**
TM18067** TM18069** TM18070** TM18071** TM18073**
TM18075** TM18076** TM18077**
89.9 TM18080**
91.3 TM18086 TM18088 TM18091 TM18092 TM18093
TM18094 TM18095 TM18096 TM18097
92.5 TM18103 TM18104 TM18107 TM18109 TM18110
TM18115
92.8 TM18138 TM18141 TM18143 TM18151 TM18152
TM18153
94.2 TM18157 TM18161 TM18168 TM18170 TM18171
TM18172 TM18176 TM18178 TM18180 TM18181
TM18182
98.3 TM18203 TM18205
98.9 TM18209
100.0 TM18224 TM18225
100.3 TM18226 TM18227 TM18228 TM18230 TM18231
TM18232 TM18233 TM18234
101.8 TM18237 TM18239
103.2 TM18242 TM18244 TM18247 TM18252 TM18256
TM18257 TM18263 TM18265 TM18266 TM18267
TM18268
103.5 TM18271
104.7 TM18276 TM18277 TM18278 TM18279
105.5 TM18281 TM18282 TM18316 TM18317 TM18320
TM18321 TM18322
106.4 TM18343
106.7 TM18345 TM18346
107.0 TM18367 TM18385 TM18386 TM18387
110.3 TM18393
112.4 TM18399 TM18400 TM18403 TM18405 TM18406
TM18407 TM18408
113.9 TM18413
114.2 TM18414 TM18416 TM18417 TM18418
118.9 TM18467
119.2 TM18470
122.0 TM18483 TM18484
0.0 TM58910 TM58914 TM58915 TM58916 TM58919
TM58920 TM58921
2.4 TM58941 TM58943
2.7 TM58944
3.8 TM58946 TM58947 TM58948 TM58949 TM58950
TM58951 TM58952
4.4 TM58954 TM58955 TM58956 TM58973
5.6 TM59013
6.1 TM59014 TM59016 TM59017 TM59018 TM59019
TM59021
6.7 TM59025 TM59026 TM59027 TM59028 TM59029
11.1 TM59039 TM59040 TM59042
11.4 TM59044 TM59045
11.7 TM59050 TM59051
12.8 TM59054
14.0 TM59057 TM59058 TM59059 TM59060
14.9 TM59066
17.6 TM59068 TM59069 TM59070 TM59072 TM59073
17.9 TM59085
18.4 TM59090
23.2 TM59105
23.5 TM59113 TM59115 TM59118 TM59119 TM59124
TM59126 TM59131 TM59132 TM59146 TM59147
25.0 TM59148
25.3 TM59153 TM59154 TM59156 TM59159 TM59162
TM59163 TM59166 TM59174 TM59177
26.4 TM59181 TM59185
29.1 TM59189
30.0 TM59190 TM59191 TM59192 TM59193
30.6 TM59194 TM59195 TM59200 TM59201
31.4 TM59210 TM59211 TM59215 TM59220 TM59221
TM59222 TM59223 TM59225
31.7 TM59228 TM59230 TM59231 TM59233 TM59242
TM59243 TM59244 TM59250 TM59253 TM59255
32.3 TM59260 TM59262 TM59265 TM59266 TM59267
TM59268
32.6 TM59270 TM59272 TM59274
33.7 TM59275
38.5 TM59279** TM59280**
38.8 TM59281 TM59282 TM59283 TM59284 TM59285
39.7 TM59287**
43.7 TM59652****
44.3 TM59653**** TM59655**** TM59656**** TM59664****
45.5 TM59673**** TM59674**** TM59676**** TM59677**** TM59678****
TM59683*** TM59684*** TM59688*** TM59689*** TM59692***
45.7 TM59693*** TM59700*** TM59703*** TM59705*** TM59706***
TM59707***
48.5 TM59730*** TM59745***
49.3 TM59754** TM59759**
50.2 TM59760***
50.5 TM59766*** TM59773*** TM59775*** TM59777*** TM59779***
61.1 TM59904**** TM59906****
62.9 TM59981****** TM59984****** TM59986****** TM59992******
TM59996******* TM59998******* TM59999******* TM60000******* TM60001*******
63.8 TM60005******* TM60007******* TM60008******* TM60009******* TM60011*******
TM60013******* TM60015******* TM60016*******
64.3 TM60017*******
TM60018******* TM60019******* TM60021******* TM60023******* TM60026*******
TM60028******* TM60031******* TM60032******* TM60033******* TM60036*******
TM60037******* TM60038******* TM60039******* TM60040******* TM60044*******
TM60047******* TM60048******* TM60049******* TM60052******* TM60054*******
TM60055******* TM60056******* TM60057******* TM60059******* TM60060*******
TM60062******* TM60063******* TM60064******* TM60068******* TM60069*******
TM60070******* TM60075******* TM60076******* TM60079******* TM60080*******
TM60081******* TM60082******* TM60090******* TM60091******* TM60094*******
TM60096******* TM60097******* TM60098******* TM60100******* TM60102*******
TM60104******* TM60105******* TM60106******* TM60109******* TM60110*******
64.9 TM60111******* TM60112******* TM60113******* TM60114******* TM60115*******
TM60117******* TM60118******* TM60119******* TM60120******* TM60122*******
TM60124******* TM60126******* TM60127******* TM60128******* TM60131*******
TM60132******* TM60133******* TM60135******* TM60136******* TM60137*******
TM60138******* TM60139******* TM60140******* TM60141******* TM60142*******
TM60144******* TM60145******* TM60146******* TM60166******* TM60184*******
TM60229******* TM60255******* TM60304******* TM60370******* TM60386*******
TM60433******* TM60465******* TM60510******* TM60513******* TM60585*******
TM60608******* TM60661******* TM60679******* TM60804******* TM60814*******
TM60945******* TM60972******* TM61090******* TM61210******* TM61277*******
TM61552*******
70.9 TM62635******
81.2 TM62916****** TM62918****** TM62919******
81.7 TM62920***** TM62921***** TM62922*****
82.0 TM62923******
87.9 TM62931** TM62934** TM62935** TM62936** TM62938**
88.5 TM62940
89.1 TM62941 TM62942 TM62944
90.8 TM62949 TM62951 TM62954
98.3 TM63037 TM63042
98.6 TM63052 TM63053 TM63054
TM63055 TM63056 TM63057 TM63059 TM63066
99.2 TM63074 TM63079 TM63081 TM63084 TM63085
TM63087 TM63089 TM63092 TM63095 TM63098
TM63104 TM63108
99.8 TM63112
100.6 TM63113
100.9 TM63114 TM63116 TM63125 TM63135 TM63136
TM63138 TM63139 TM63143 TM63144 TM63148
TM63149
101.2 TM63153
101.5 TM63155
102.0 TM63157 TM63158 TM63159 TM63160 TM63161
TM63162 TM63163 TM63164 TM63166 TM63167

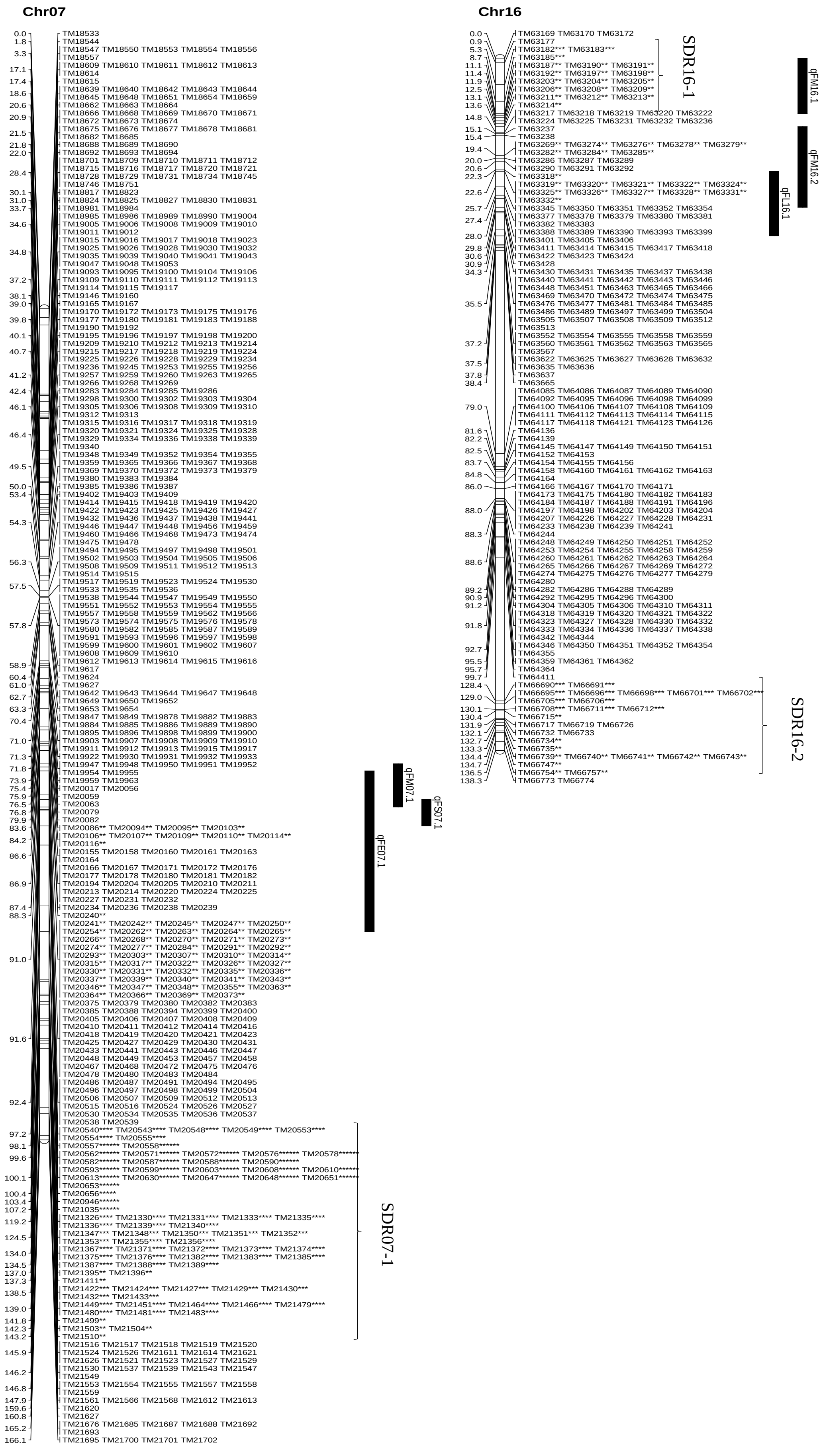

Chr07
Chr16
SDR07-1
SDR16-1
SDR16-2
qFM07.1
qFS07.1
qFE07.1
qFM16.1
qFS16.1
qFU16.1
qFM16.2
qFS16.2
qFL16.1

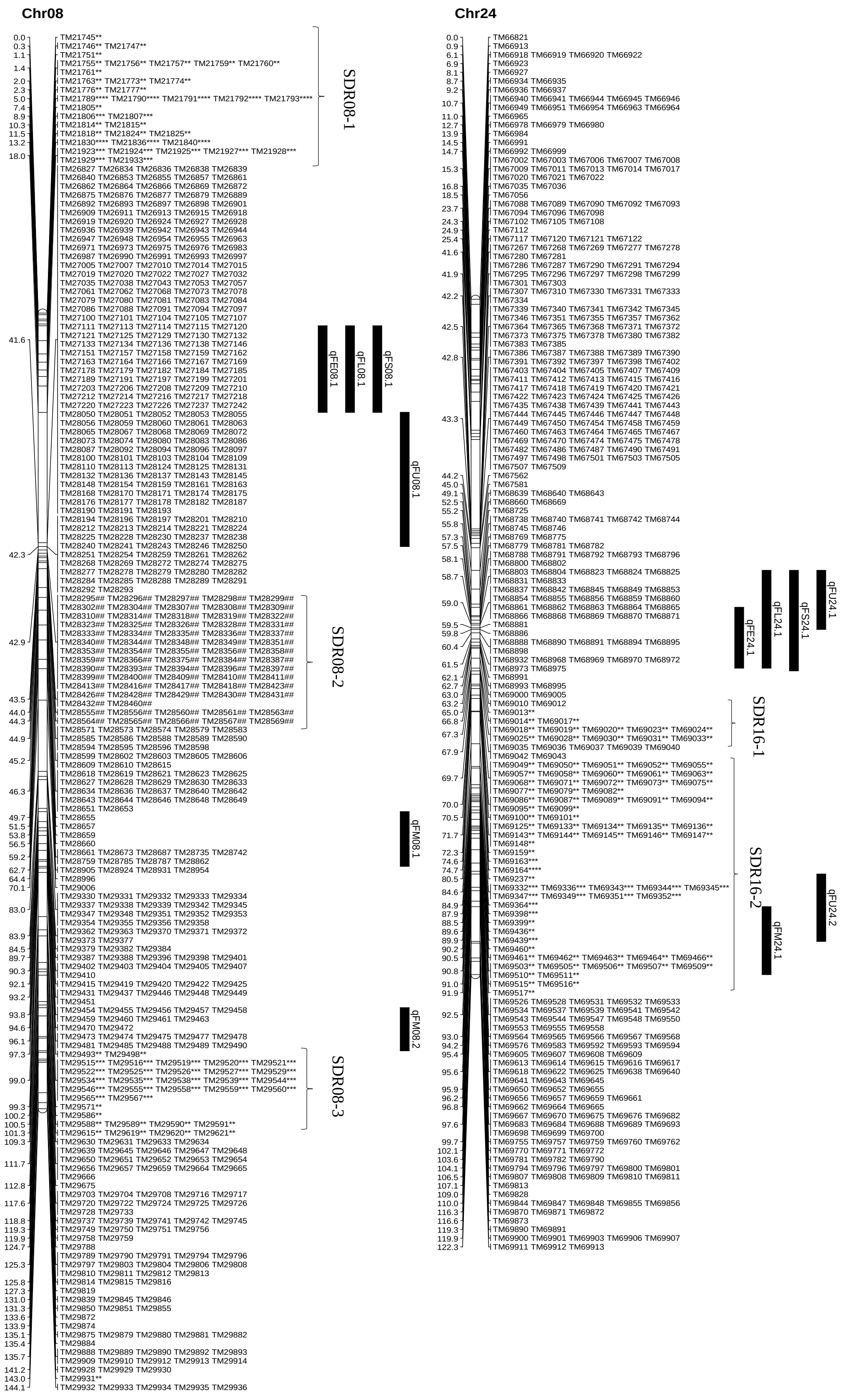

Chr08
Chr24
SDR08-1
SDR08-2
SDR08-3
qFE08.1
qFL08.1
qFS08.1
qFU08.1
qFM08.1
qFM08.2
SDR16-1
SDR16-2
qFE24.1
qFL24.1
qFS24.1
qFU24.1
qFU24.2
qFM24.1

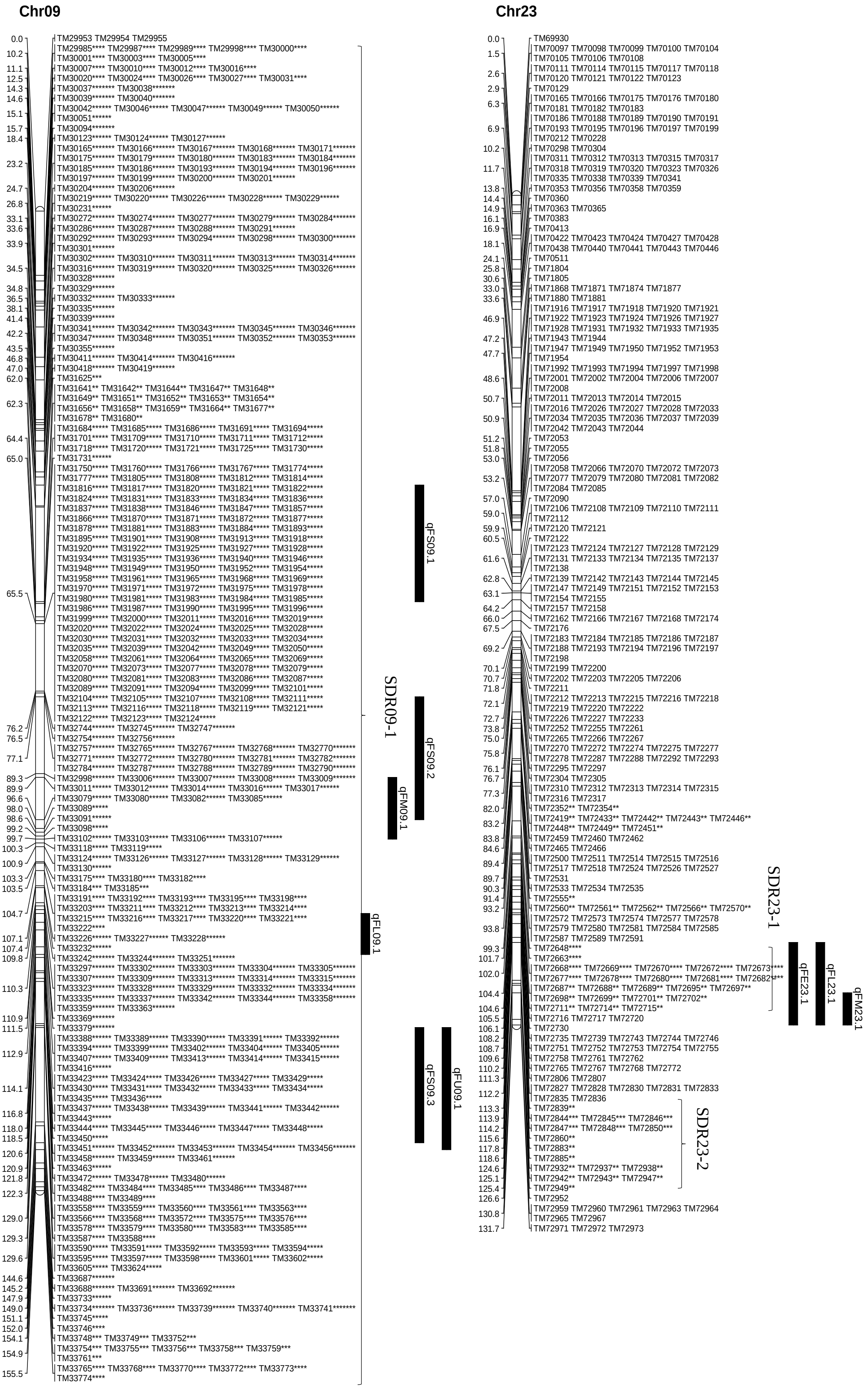
Chr09
Chr23
SDR09-1
qFS09.1
qFS09.2
qFM09.1
qFL09.1
qFS09.3
qFU09.1
SDR23-1
SDR23-2
qFE23.1
qFL23.1
qFM23.1

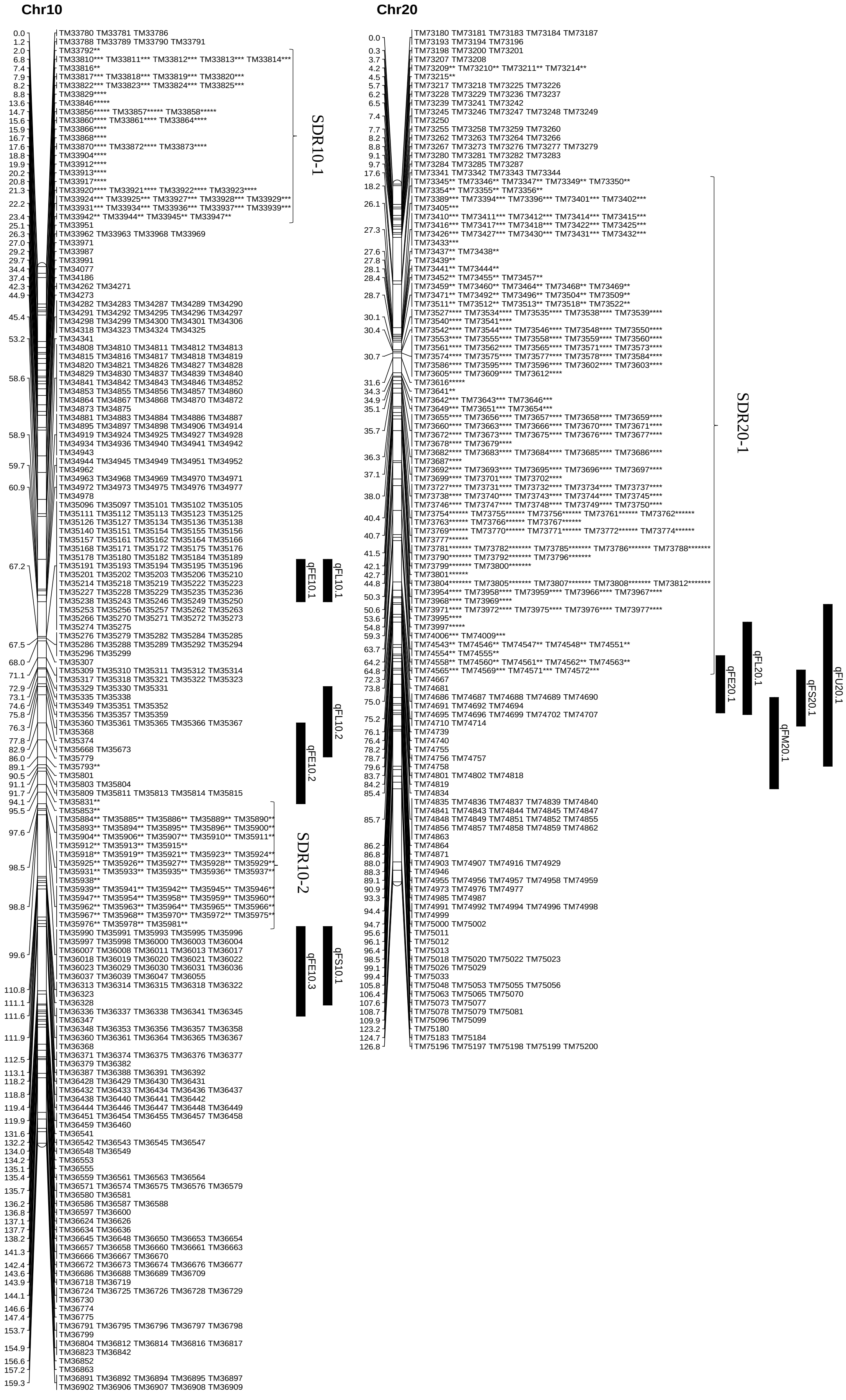
Chr10
Chr20
SDR10-1
SDR10-2
SDR20-1
qFL10.1
qFE10.1
qFL10.2
qFE10.2
qFS10.1
qFE10.3
qFE20.1
qFL20.1
qFS20.1
qFM20.1
qFU20.1

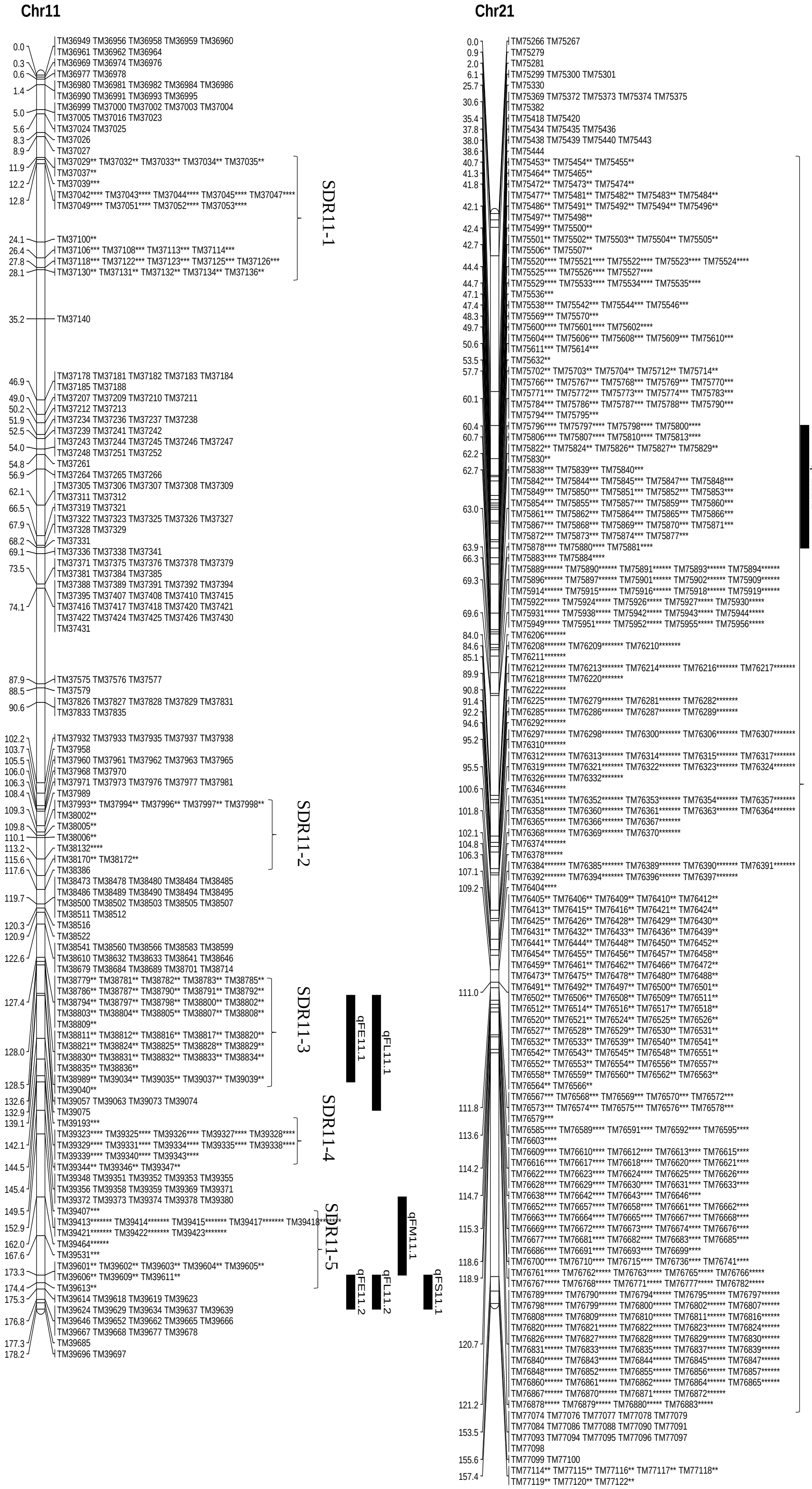

Chr11
Chr21
SDR11-1
SDR11-2
SDR11-3
SDR11-4
SDR11-5
qFE11.1
qFL11.1
qFM11.1
qFE11.2
qFL11.2
qFS11.1
SDR21-1
qFM21.1
qFE21.1
qFL21.1
qFS21.1
qFU21.1
qFU21.2
qFL21.2

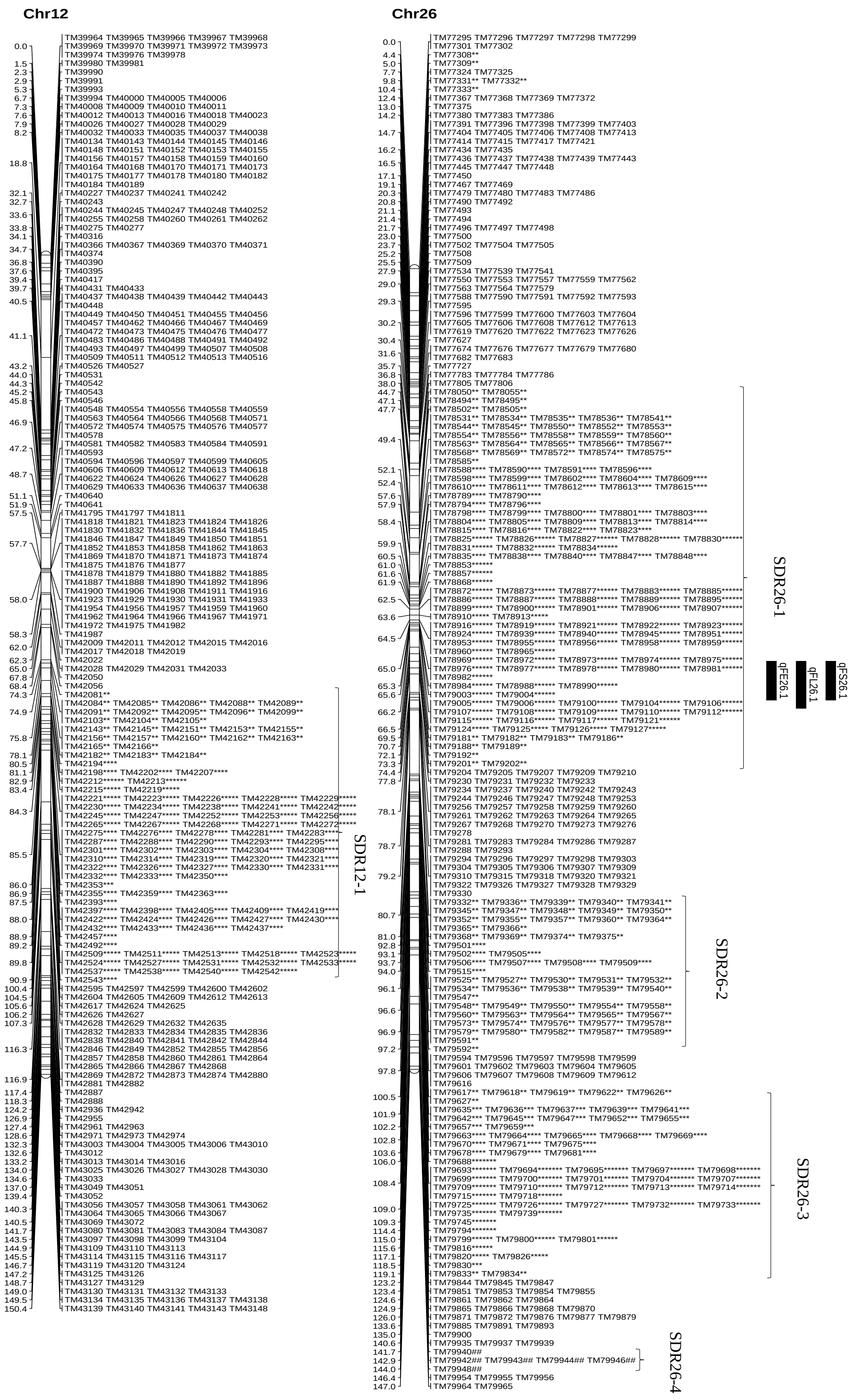
Chr12
Chr26
SDR12-1
SDR26-1
SDR26-2
SDR26-3
SDR26-4
qFS26.1
qFL26.1
qFE26.1

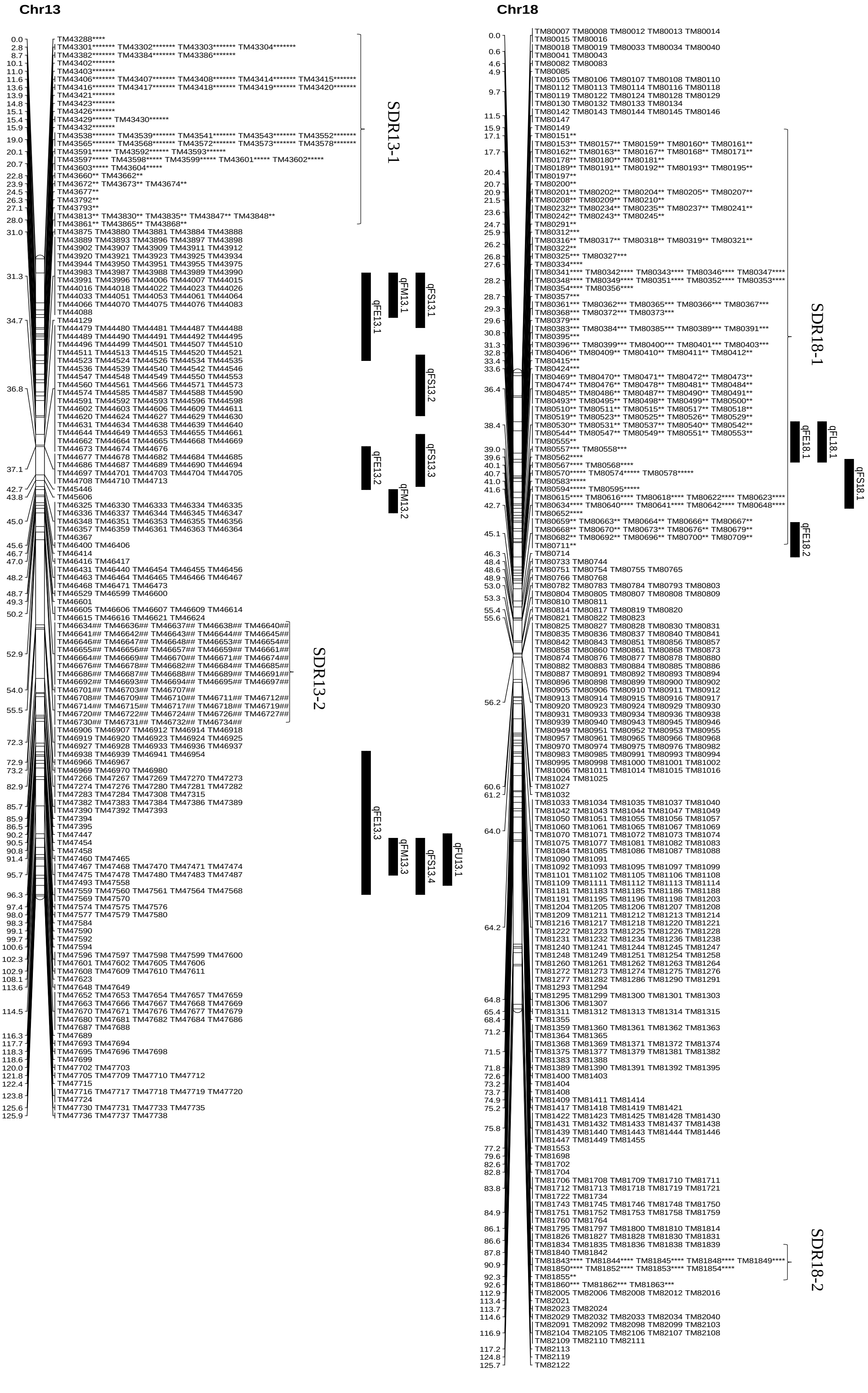

Figure S1. Genetic maps and QTL for fiber quality in RIL population. Map distances were given in

centimorgans (cM). Markers distorted favored toward Yumian 1 were followed by asterisks with the level of ** P < 0.05, *** P < 0.01, **** P < 0.005, ***** P< 0.001, ****** P < 0.0005, ******* P < 0.0001 according the standard of Joinmap 4.0, the replaced sign (#) distorted favored toward Acala Maxxa. Bars along the genetic map indicated the confidence QTL interval. QTL were shown as FL for fiber length, FU for fiber uniformity, FS for fiber strength, FE for fiber elongation, and FM for fiber micronaire.
